# Meta-analysis on the efficacy and safety of Guanxin Shutong capsule in the treatment of angina pectoris of coronary heart disease

**DOI:** 10.3389/fendo.2025.1534752

**Published:** 2025-06-17

**Authors:** Yaqin Wang, Muchen Zhang, Yan Zhang, Wenhao Yin, Shujun Zhao, Wenjun Mao, Xuewei Wang

**Affiliations:** ^1^ First Clinical College, Liaoning University of Traditional Chinese Medicine, Shenyang, China; ^2^ Department of Chinese Medicine, Shenyang Anning Hospital, Shenyang, China; ^3^ Expert Clinic, Traditional Chinese Medicine Hall, Affiliated Hospital of Liaoning University of Traditional Chinese Medicine, Shenyang, China; ^4^ Department of Obstetrics, Shenyang Women and Infants Hospital, Shenyang, China

**Keywords:** GXST capsule, coronary heart disease, meta-analysis, systematic review, traditional Chinese medicine

## Abstract

**Objective:**

This study aims to summarize all single clinical studies of Guanxin Shutong (GXST) capsule combined with Western medicine in the treatment of coronary heart disease angina pectoris and to systematically evaluate its efficacy and safety.

**Methods:**

This study adhered to the Preferred Reporting Items for Systematic Reviews and Meta-Analyses (PRISMA) guidelines. Chinese and English databases were searched to collect the randomized controlled trials (RCTs) of GXST capsule combined with conventional Western medicine in the treatment of patients with angina pectoris of coronary heart disease and to extract the data. Cochrane Risk of Bias Tool was used to evaluate literature quality, and RevMan5.3 software was used to evaluate the outcome indicators, such as total effective rate of angina pectoris, frequency of angina pectoris, duration of angina pectoris, total effective rate of electrocardiogram (ECG), lipid level, inflammatory factor level, hemorheology, cardiac function, and adverse reactions, and to assess publication bias.

**Results:**

A total of 27 RCTs with 3440 cases were identified. The results showed that the combined use of GXST capsule was more effective in terms of total effective rate of angina pectoris, frequency of angina pectoris, duration of angina pectoris, and total effective rate of ECG. In addition, the combined use of GXST capsule had more advantages in reducing total cholesterol, low-density lipoprotein cholesterol, interleukin-6, tumor necrosis factor–α, high-sensitivity C-reactive protein, and whole-blood viscosity and increasing left ventricular ejection fraction and high-density lipoprotein cholesterol. However, for triglyceride and interleukin-1, there were two different results before and after the sensitivity analysis, which were attributed to the quality of the included literature. In terms of plasma viscosity and adverse reactions, after excluding the literature with large heterogeneity, sensitivity analysis indicated that the combined use of GXST capsule was helpful to reduce plasma viscosity and adverse reactions.

**Conclusions:**

GXST capsule combined with conventional Western medicine has better efficacy and safety in the treatment of angina pectoris of coronary heart disease compared with Western medicine alone. However, our study still has some limitations. Thus, more standardized RCTs are needed in future studies to verify the conclusions, and longer follow-up periods need to be designed to explore the long-term efficacy.

## 1 Introduction

Coronary atherosclerotic heart disease, also known as coronary heart disease (CHD), is a heart disease that causes varying degrees of injury due to coronary artery stenosis or occlusion caused by coronary atherosclerosis, leading to myocardial ischemia, hypoxia, or necrosis (1). CHD mostly occurs in middle-aged and elderly people, more in men than in women, and predominantly in brain workers, which is a major disease that jeopardizes people’s health globally, especially in developed countries (2). In recent years, Western medicine has made rapid development in the diagnosis and treatment of CHD, but it has not yet achieved satisfactory results in some aspects (3–5). More and more patients with CHD are seeking complementary and alternative treatments.

Chinese medicine has made great progress in the prevention and treatment of CHD by utilizing its own characteristics and combining with Western medicine. The application of traditional Chinese medicine (TCM) in the treatment of CHD has a long history in China. Under the theoretical system of TCM syndrome differentiation and treatment, Chinese people combine various botanical drugs into prescriptions, which have rich theoretical connotation and therapeutic experience in the treatment of CHD (6, 7). Commercial Chinese polyherbal preparation (CCPP) Guanxin Shutong (GXST) capsule is a new drug developed by combining the theory of ethnic characteristics of Mongolian medicine with clinical practice, which is mainly used for the treatment of angina pectoris, as noted in Pharmacopoeia of the people’s Republic of China (2020) (https://ydz.chp.org.cn/#/item?bookId=1&entryId=1743) and drug instructions (buchang.com/newZBT/pages/product/productDetail.html). GXST capsule is composed of five botanical drugs, including *Choerospondias axillaris* (Roxb.) B.L. Burtt & A.W. Hill (Anacardiaceae; Choerospondiatis fructus), *Salvia miltiorrhiza* Bunge (Lamiaceae; Salviae miltiorrhizae radix et rhizoma), *Syzygium aromaticum* (L.) Merr. & L.M. Perry (Myrtaceae; caryophylli foris aetheroleum), *Camphora officinarum* Nees (Lauraceae; camphor leaves), and *Bambusa textilis* McClure (Poaceae; bambusae concretio silicea) (8). In Traditional Chinese Medicine Systems Pharmacology Database and Analysis Platform (https://www.tcmsp-e.com/#/database), the screening criteria was set to Oral Bioavailability≥30% (OB≥30%), Caco-2 Permeability≥ -0.4 (Caco-2≥-0.4) (HL≥4), Drug Likeness≥0.18 (DL≥0.18), and Half-Life≥4 (9) and the botanical drug metabolites were inquired. The composition is detailed in **Supplementary File S1**. In the more than 20 years since its launch, there have been multiple evidence-based medical studies on the combination of GXST capsule and Western medicine for the treatment of CHD angina pectoris, for which no systematic evaluation of safety and clinical efficacy has been conducted currently. Meta-analysis is a high-level evaluation evidence in clinical studies. Therefore, the purpose of this paper is to summarize all the individual clinical studies of GXST capsule combined with Western medicine in the treatment of angina pectoris of CHD and to systematically evaluate its efficacy and safety, so as to provide strong evidence for clinical decision-making by clinical workers and provide clear support for the promotion of CCPP and simple preparations.

## 2 Methods

### 2.1 Search strategy

This study adhered to the PRISMA guidelines (10, 11). Wanfang Data, China National Knowledge Infrastructure (CNKI), China Biology Medicine disc (CBM disc), China Science and Technology Journal Database (VIP), and foreign databases, including Cochrane Library, EMBASE, and PubMed, were searched from database building to 21 December 2023, with the search terms “Guanxinshutong capsule,” “GXST,” “Coronary disease,” “Angina Pectoris,” “CHD,” “chest pain,” etc. Chinese databases were searched with the translation of the above terms. Review articles, review references, and conference abstracts were also reviewed to avoid missing potential studies that met the inclusion criteria.

### 2.2 Selection criteria

The following criteria were pre-specified according to the PICO model (12).

1) Study subjects: All patients with angina pectoris of CHD diagnosed by modern medicine, regardless of age, sex, race, clinical type, and course of disease.
2) Interventions: The control group received guideline-conforming therapy tailored to angina classification (with or without placebo): For stable angina patients:β-blocker therapy, long-acting nitrate, calcium channel blocker as needed, high-intensity statin; for unstable angina patients: dual antiplatelet loading (aspirin 300 mg + ticagrelor 180 mg on admission), anticoagulation, intravenous nitroglycerin titration, high-dose statin (atorvastatin of 80 mg nocte); all control patients received sublingual nitroglycerin (0.5 mg prn) as rescue medication. Dose adjustments were made per 2023 European Society of Cardiology (ESC) guidelines based on blood pressure/heart rate monitoring. The treatment group received the aforementioned stratified therapy combined with GXST capsules (0.9g, tid), maintaining identical conventional treatment protocols between groups.
3) Study type: All published randomized controlled trials (RCT).
4) Outcome: The study included at least one of the following outcome indicators: ① total effective rate of angina pectoris, ② frequency of angina pectoris, ③ duration of angina pectoris, ④ total effective rate of electrocardiogram (ECG), ⑤ lipid level, ⑥ inflammatory factor level, ⑦ hemorheology, ⑧ heart function: left ventricular ejection fraction (LVEF), ⑨ adverse reactions. Among them, ① to ④ were the main efficacy indicators, ⑤ to ⑧ were the secondary efficacy indicators, and ⑨ was the safety evaluation indicators.

Exclusion criteria: 1) Studies that could not be traced back to the full text; 2) studies such as animal experiments, consensus, reviews, case reports, and systematic reviews; and 3) duplicate published studies.

### 2.3 Literature screening and data extraction

First, the databases were searched by two reviewers independently and imported with Note Express. software. The titles and abstracts were screened after duplicate documents were removed to exclude the literature unrelated to the study topic. After that, full texts were evaluated to eliminate non-compliant literature according to the inclusion and exclusion criteria. When the two reviewers have different opinions or doubts, a third-party reviewer can be requested to conduct screening and discussion to make a selection of the literature. After inclusion of eligible literature, data extraction was performed, including: characteristics of the literature (first author and year of publication), type of study design, sample size, demographic characteristics, interventions, outcome indicators, adverse reactions, and other useful information. Data were cross-checked and input.

### 2.4 Quality evaluation

The quality of included studies was independently evaluated from six aspects according to the method described in the Cochrane Handbook for Systematic Reviews of Interventions (13), including random sequence generation, allocation concealment, blinding of participants and personnel, blinding of outcome assessment, incomplete outcome data, selective reporting, and other bias, with ratings of “low risk of bias,” “high risk of bias,” and “unclear risk of bias.” Quality evaluation was conducted by two post-graduates. Disagreements, if any, would be resolved through discussion, and, if necessary, the original authors could be contacted to verify potential risks of bias. The risk assessment of subjective outcomes and objective outcomes was assessed separately using RoB2 (14).

### 2.5 Statistics and analysis

RevMan5.3 software was used for statistics. Measurement data were represented by mean difference (MD) or standard mean difference (SMD), and binary classification data were described by relative risk (RR), all of which were expressed as 95% confidence interval (CI), and *P <* 0.05 was statistically significant. Heterogeneity results were expressed as I^2^. I^2^ ≤ 50% indicated that the heterogeneity of each study was acceptable, and fixed-effects model was used for analysis. I^2^ > 50% indicated large heterogeneity of the study, and sensitivity analysis or subgroup analysis was used to identify the source of heterogeneity. If there was still significant heterogeneity, random-effects model was used to combine the effect size. When the number of studies was not less than 10, the funnel plot was used to check for the presence of bias.

## 3 Results

### 3.1 Literature retrieval

**Figure 1:**
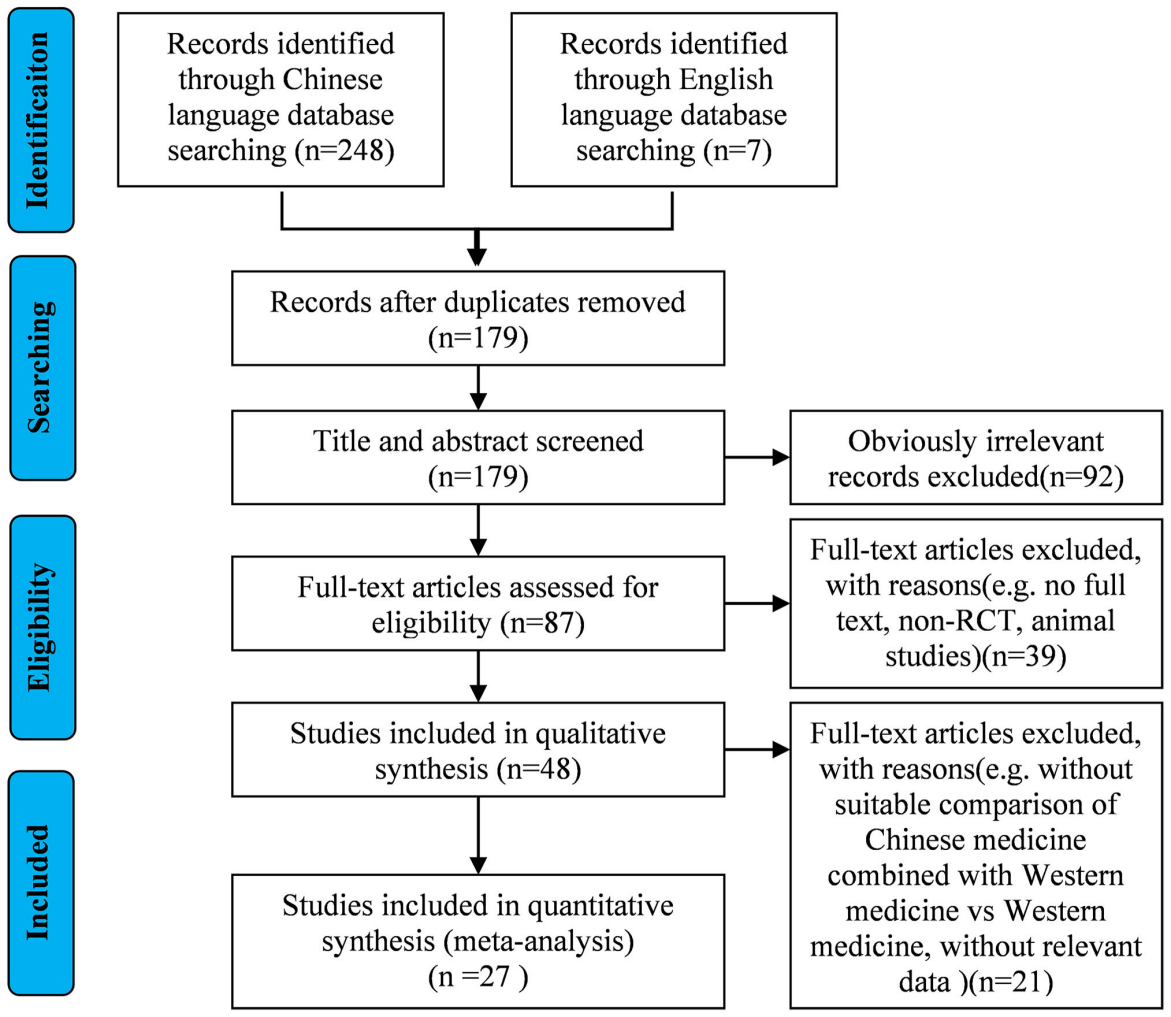
Literature screening process.

A total of 248 records were retrieved from Chinese databases, with an additional 7 identified from English sources. Following deduplication procedures, 76 duplicate entries were eliminated. During the title/abstract screening phase, 92 articles were excluded as irrelevant. The remaining 87 publications underwent full-text eligibility assessment based on predetermined inclusion/exclusion criteria. Ultimately, 27 studies met the requirements for final inclusion. The complete literature selection process is detailed in **Figure 1**.

### 3.2 Characteristics of the included studies

All studies were RCTs, of which 1 (15) was from an English database and 26 from a Chinese database. For intervention, conventional Western medicine [including aspirin, beta-blockers, calcium channel blocker (CCB), statins, and angiotensin-converting enzyme inhibitor (ACEI)/angiotensin receptor blocker (ARB)] was adopted in the control group, whereas GXST capsule (0.9 g, tid) combined with conventional Western medicine was administrated in the treatment group. The duration of the study ranged from 4 to 12 weeks (**Table 1**).

**Table 1:** General information on included studies.

First author, publication year	Sample size (T/C)	Diagnosis	Gender (male/female)	Age (mean ± SD or range in years)	Duration of AP (mean ± SD or range in years)	Treatment duration (weeks)	Outcomes
R.Y. Cai,, 2022 16	62/62	NT	T: 43/19 C: 41/21	T: 62.69 ± 4.8 C: 62.63 ± 4.71	T: - C: -	4	①, ②, ③, ⑤, ⑥, and ⑨
X.Y. Cao, 2013 17	42/42	NT	T: 29/13 C: 31/11	T: 61.3 ± 5.2 C: 60.1 ± 6	T: 7.8 ± 3.7 C: 7.2 ± 4.3	4	④
Y.J. Chen, 2016 18	50/50	UAP	T: 21/29 C: 24/26	T: 40~65 C: 41~65	T: 2~11 C: 2~11	4	①, ②, ③, and ⑥
W.H. Gou, 2020 19	150/150	UAP	T: 76/74 C: 72/78	T: 53.81 ± 8.12 C: 52.48 ± 7.29	T: - C: -	4	①, ②, ③, ⑥, ⑦, and ⑨
D.Q. Guo, 2013 20	42/40	UAP	T: 23/19 C: 22/18	T: 63.42 ± 17.73 C: 62.21 ± 16.34	T: 5.41 ± 3.27 C: 5.52 ± 3.43	4	④
Y.Y. Ji,, 2017, 21	43/42	SAP	T: 20/23 C: 24/18	T: 45.12 ± 0.61 C: 46.12 ± 0.41	T: 3.03 ± 0.31 C: 3.11 ± 0.21	8	①
Q.F. Jia, 2023, 22	57/57	SAP	T: 31/26 C: 34/23	T: 60.05 ± 5.91 C: 59.42 ± 6.28	T: 12.24 ± 1.44 C: 12.17 ± 1.52	12	①, ⑤, and ⑥
X.B. Li, 2014 23	34/34	NT	T: 27/7 C: 25/9	T: 77.5 ± 4.4 C: 75.2 ± 5.4	T: 9.4 ± 1.4 C: 8.7 ± 1.5	6	①
Y. Li, 2019 15	143/144	SAP	T: 73/70 C: 82/62	T: 56.6 ± 8.1 C: 55.3 ± 8.3	T: - C: -	4	①, ②, and ⑨
B. Liang, 2015 24	43/43	UAP	T: 27/16 C: 25/18	T: 61.8 ± 4.2 C: 61.1 ± 5.6	T: - C: -	4	①
Y.C. Lin, 2016 25	39/39	UAP	T: 21/18 C: 23/16	T: 65.13 ± 7.04 C: 64.81 ± 6.97	T: - C: -	4	①
S.S. Liu, 2019 26	66/66	SAP	T: 37/29 C: 39/27	T: 69.5 ± 5.7 C: 69.6 ± 5.2	T: 2.3 ± 0.2 C: 2.1 ± 0.3	6	① and ④
S.F. Luo, 2021 27	150/150	SAP	T: 68/82 C: 66/84	T: 58.7 ± 3.9 C: 59.4 ± 3.5	T: - C: -	8	①, ⑤, and ⑥
L.J. Miao, 2017 28	44/43	UAP	T: 27/17 C: 25/18	T: 55.9 ± 7.29 C: 56.88 ± 7.32	T: 5.1 ± 1.3 C: 4.17 ± 1.21	4	②, ③, ④, and ⑨
L. Pan, 2022 29	54/54	SAP	T: 33/21 C: 31/23	T: 57.92 ± 3.96 C: 58.69 ± 4.18	T: 3.19 ± 0.32 C: 3.14 ± 0.28	4	①, ⑥, ⑦, and ⑨
S.H. Ren, 2017 30	41/40	NT	T: 25/16 C: 23/17	T: 66.05 ± 8.48 C: 65.48 ± 8.37	T: - C: -	8	①, ④, ⑦, and ⑧
H. Shi, 2022 31	48/48	SAP	T: 26/22 C: 25/23	T: 66.18 ± 1.25 C: 62.97 ± 1.20	T: 2.78 ± 1.01 C: 2.9 ± 1.05	4	①, ②, ③, ⑤, ⑥, and ⑧
J. Shi, 2018 32	41/41	SAP	T: 21/20 C: 20/21	T: 60.4 ± 11.1 C: 59.2 ± 12.4	T: 6.8 ± 3.6 C: 7.1 ± 3.8	12	①, ②, ③, ⑤, and ⑥
J.H. Song, 2016 33	81/81	NT	T: 46/35 C: 56/25	T: 59.45 ± 5.21 C: 61.23 ± 4.97	T: 4.23 ± 1.26 C: 5.12 ± 0.96	6	①
G. Wang, 2021 34	40/40	SAP	T: 24/16 C: 22/18	T: 43.42 ± 7.39 C: 43.68 ± 7.45	T: 4.41 ± 1.16 C: 4.58 ± 1.24	4	①, ④, and ⑥
Y. Wang, 2015 35	40/40	SAP	T: 26/14 C: 17/13	T: 59.9 ± 7.4 C: 60.4 ± 7.6	T: - C: -	4	①, ④, and ⑥
Z.J. Wu, 2015 36	181/181	SAP	T: 114/67 C: 100/81	T: 63 ± 1 C: 62.5 ± 1	T: 6.11 ± 1.67 C: 6.01 ± 1.71	6	①, ④, and ⑤
N. Yang, 2018 37	30/30	UAP	T: 19/11 C: 18/12	T: 65.45 ± 1.23 C: 66.45 ± 1.98	T: 4.12 ± 1.34 C: 4.23 ± 1.05	8	①, ⑥, and ⑨
Z.C. You, 2021 38	30/30	NT	T: 16/14 C: 18/12	T: 57.43 ± 3.34 C: 56.52 ± 3.51	T: - C: -	4	① and ⑨
J.S. Zhao, 2015 39 Zhao, 2015 40	40/40	UAP	T: 24/16 C: 22/18	T: 55.45 ± 8.35 C: 56.86 ± 9.04	T: 3.35 ± 1.07 C: 3.67 ± 1.58	4	① and ④
K. Zhao, 2015 40	67/65	SAP	T: 45/22 C: 51/14	T: 60 ± 9.9 C: 58.4 ± 10.1	T: - C: -	4	① and ⑨
X.J. Zhu, 2017 41	65/65	SAP	T: 38/27 C: 40/25	T: 52.8 ± 2.4 C: 52.4 ± 1.8	T: 4.9 ± 2.1 C: 4.7 ± 1.5	4	① and ⑨

T, treatment group; C, control group; AP, angina pectoris; SAP, stable angina pectoris; UAP, unstable angina pectoris; NT, not tested. Outcome: ① Total effective rate of angina pectoris; ② frequency of angina pectoris; ③ duration of angina pectoris; ④ total effective rate of ECG; ⑤ lipid level; ⑥ inflammatory factor level; ⑦ hemorheology; ⑧ heart function: LVEF; and ⑨adverse reactions.

### 3.3 Quality evaluation

**Figure 2:**
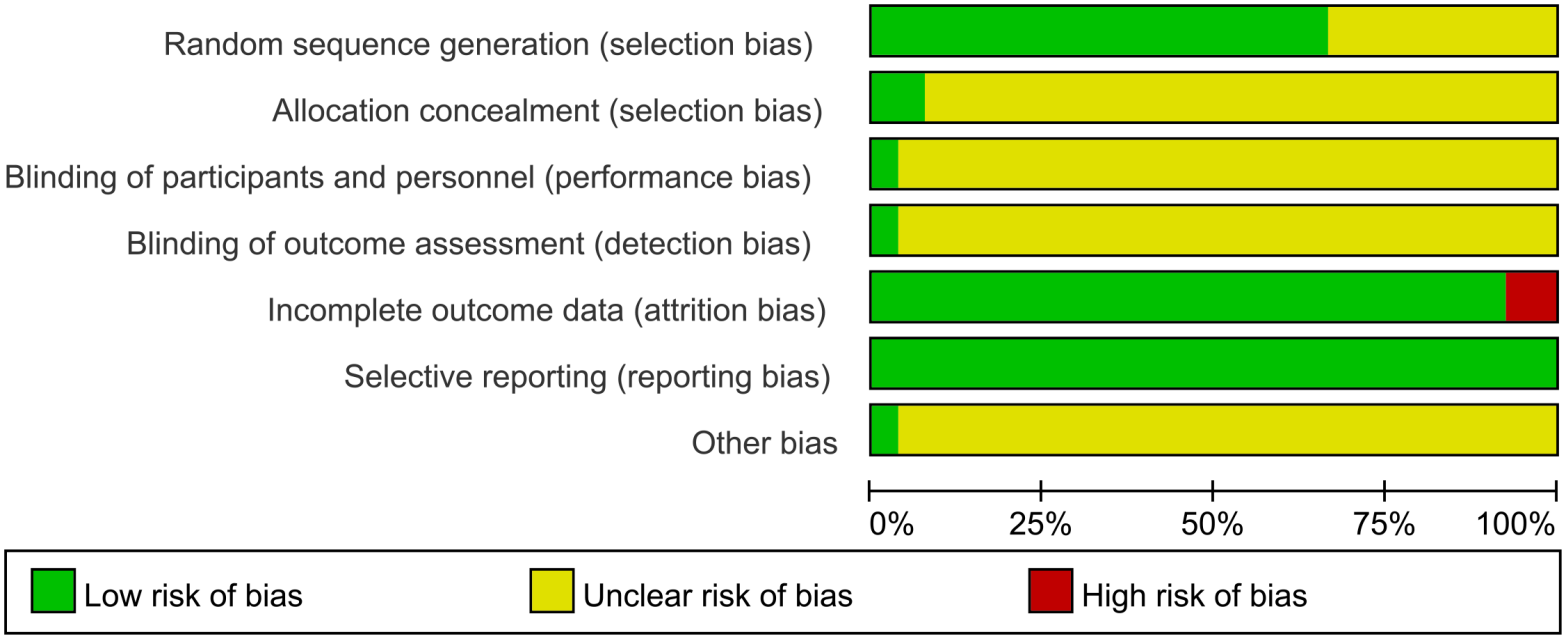
Summary of risk bias.

**Figure 3:**
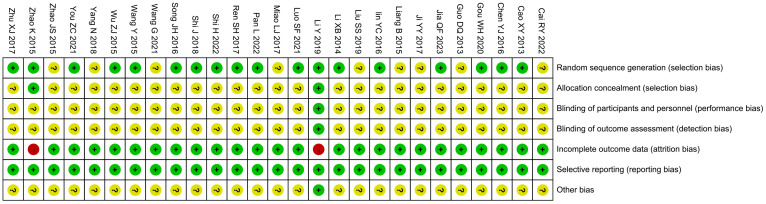
Risk of bias domain for each included study.

All studies were RCTs, of which 18 studies described the random allocation method in detail with the correct method (random number table method, computer table method, or lottery method), whereas the remaining 9 studies (16, 20, 21, 24, 26, 28, 34, 37, 39) did not describe the specific random allocation method. Two studies (15, 40) employed allocation concealment, using sealed opaque envelopes, which were rated as low risk, and the remaining studies did not mention allocation concealment. One study mentioned the use of blinding for subjects, implementers, and data analyzers and was rated as low risk (15), although it was not clear whether blinding was used in the remaining studies. Two studies (15, 40) did not complete the outcome that included all patients at the beginning of the study due to dropping out of patients and were considered high risk. All studies fully reported all pre-set outcomes. For other biased outcomes, one study (15) reported no conflict of interest and the rest were not mentioned. See **Figures 2** and **3**. The risk assessment of subjective outcomes and objective outcomes were assessed separately. They are shown in **Supplementary File S2**.

### 3.4 Meta-analysis of primary outcomes

#### 3.4.1 Total effective rate of angina pectoris

**Figure 4:**
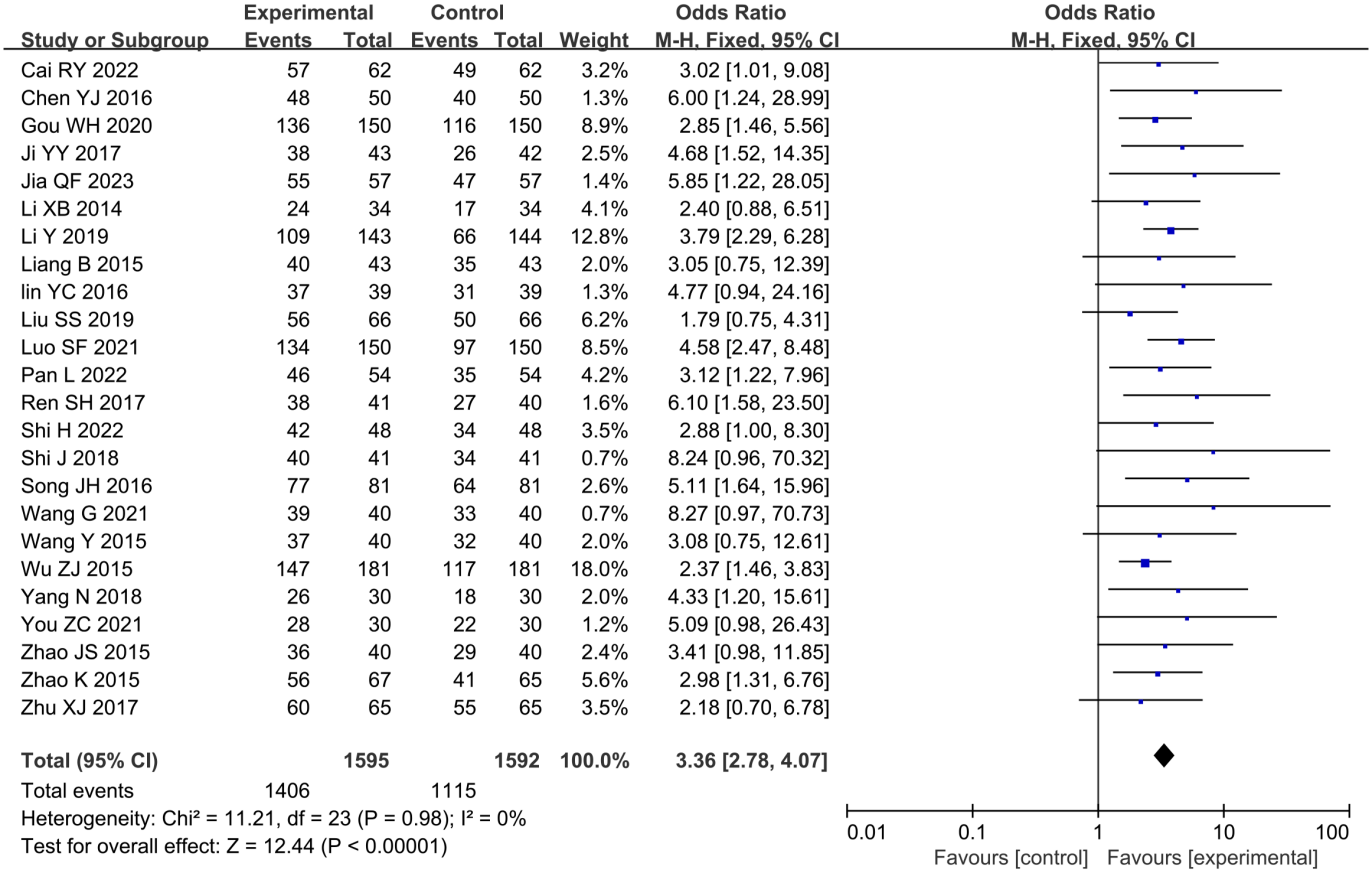
Meta-analysis results of the total effective rate of angina pectoris.

A total of 24 studies reported the total effective rate of angina pectoris, involving a total of 3,187 subjects, including 1,595 in the treatment group and 1,592 in the control group. Statistically, there was no heterogeneity (*P* = 0.98, *I* ^2^ = 0). The fixed-effects model meta-analysis demonstrated a significantly higher total effective rate in the GXST combination therapy group compared to that in conventional treatment alone [n = 3,187; OR, 3.36; 95% CI (2.78, 4.07); *P* < 0.00001] (**Figure 4**). This indicates that adjunctive GXST capsule therapy enhanced angina pectoris management when combined with guideline-directed medical therapy. According to Grading of Recommendations Assessment, Development and Evaluation (GRADE) evidence quality evaluation, the total effective rate of angina pectoris was moderate-quality evidence, as shown in **Supplementary File S3**.

#### 3.4.2 Frequency of angina pectoris

**Figure 5:**
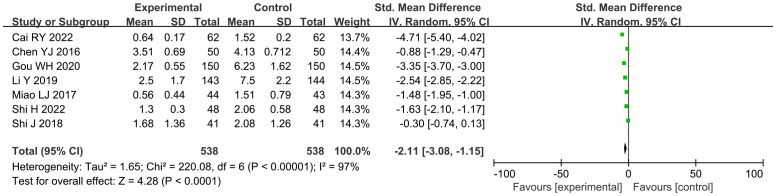
Meta-analysis results of the frequency of angina pectoris.

A total of seven studies (15, 16, 18, 19, 28, 31, 32) reported the frequency of angina pectoris, involving a total of 1,076 subjects, including 538 in both the treatment group and the control group. Statistically, there was heterogeneity (*P* < 0.00001, *I* ^2^ = 97%). Leave-one-out sensitivity analysis demonstrated that no single study disproportionately influenced the pooled effect estimate. Random-effects model meta-analysis revealed a substantial reduction in angina frequency with GXST adjuvant therapy versus guideline-based treatment alone [n = 1076; SMD, −2.11; 95% CI (−3.08, −1.15); *P* < 0.0001] (**Figure 5**). The effect magnitude suggests clinically meaningful improvement according to ESC chronic coronary syndrome management targets. According to GRADE evidence quality evaluation, the frequency of angina pectoris was moderate-quality evidence, as shown in **Supplementary File S3**.

#### 3.4.3 Duration of angina pectoris

**Figure 6:**
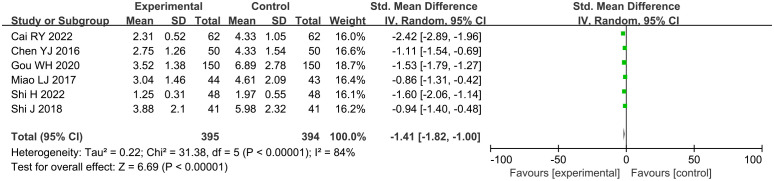
Meta-analysis results of the duration of angina pectoris.

A total of six studies (16, 18, 19, 28, 31, 32) reported the duration of angina pectoris, involving a total of 789 subjects, including 395 in the treatment group and 394 in the control group. Statistically, heterogeneity was detected (*P* < 0.00001, *I* ^2^ = 84%). Leave-one-out sensitivity analysis demonstrated that no single study disproportionately influenced the pooled effect estimate. The random-effects model was used for meta-analysis, and this analysis showed significant results [n = 789; SMD, −1.41; 95% CI (−1.82, −1.00); *P* < 0.00001] (**Figure 6**). In general, combined use of GXST capsule on the basis of conventional Western medicine reduced the duration of angina pectoris. According to GRADE evidence quality evaluation, the duration of angina pectoris was low-quality evidence, as shown in **Supplementary File S3**.

#### 3.4.4 ECG efficacy

**Figure 7:**
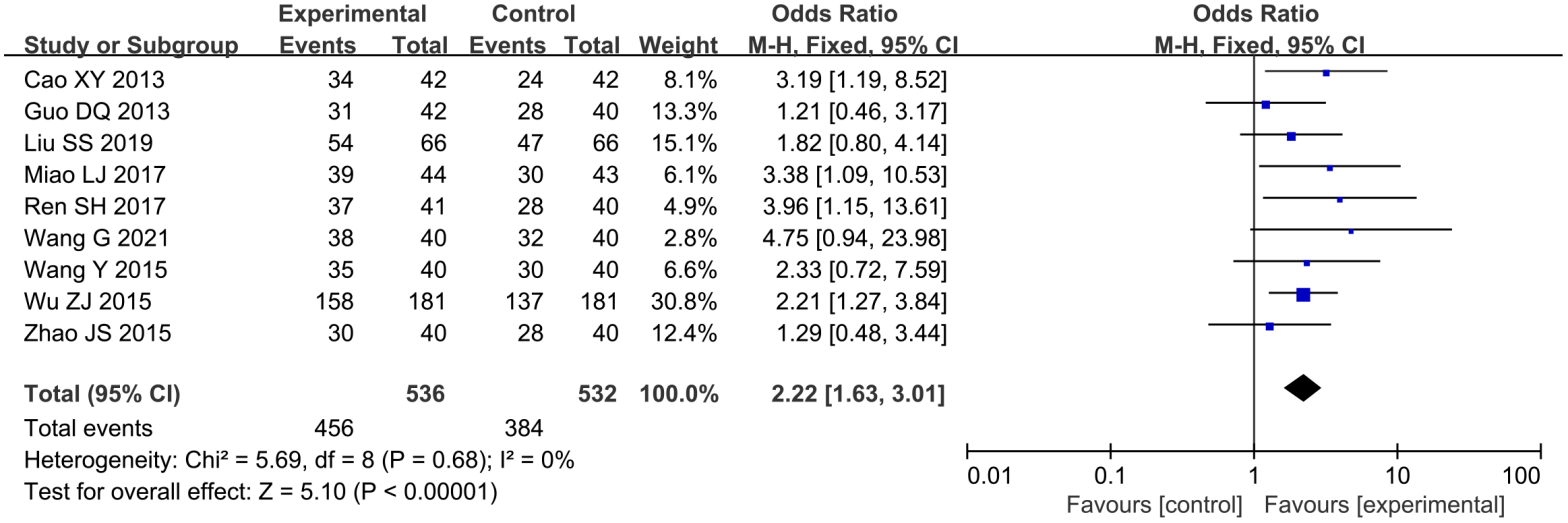
Meta-analysis results of electrocardiogram efficacy.

A total of nine studies (15, 17, 20, 28, 30, 34–36, 39) reported the efficacy of ECG, involving a total of 1068 subjects, including 536 in the treatment group and 532 in the control group. Statistically, there was no heterogeneity (*P* = 0.68, *I* ^2^ = 0). Fixed-effects model meta-analysis demonstrated significant improvement [n = 1068, OR: 2.22; 95% CI (1.62, 3.01); *P* < 0.00001] (**Figure 7**). In general, combined use of GXST capsule on the basis of conventional Western medicine increased the total effective rate of ECG. According to GRADE evidence quality evaluation, ECG efficacy was moderate-quality evidence, as shown in **Supplementary File S3**.

### 3.5 Meta-analysis of secondary outcomes

#### 3.5.1 Lipid level

##### 3.5.1.1 TC

**Figure 8:**
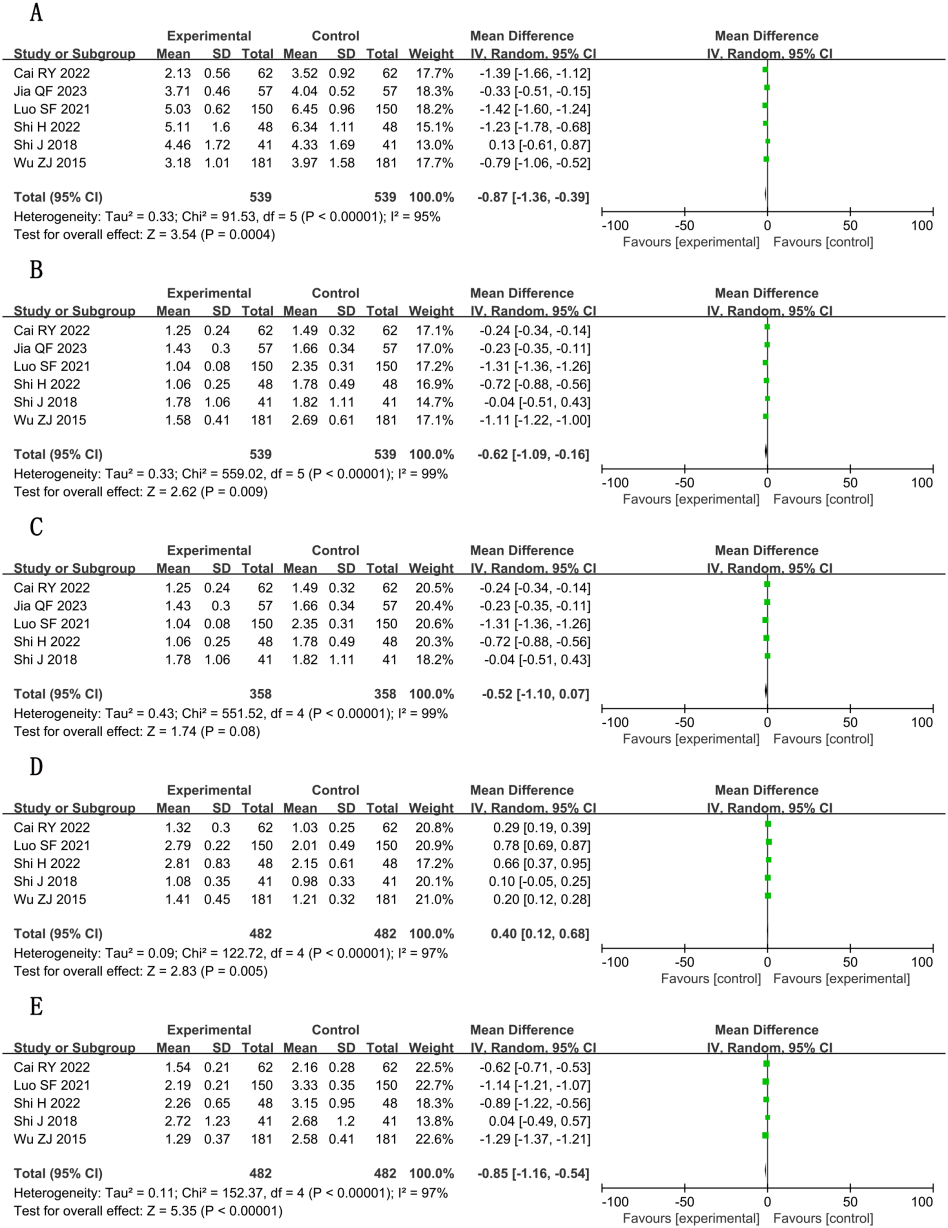
Forest plots for meta-analysis. **(A)** TC; **(B)** TG; **(C)** TG (adjusted); **(D)** HDL-C; **(E)** LDL-C.

A total of six studies (16, 22, 27, 31, 32, 36) reported total cholesterol (TC) levels, involving a total of 1,078 subjects, including 539 in both the treatment group and the control group. Statistically, there was heterogeneity (*P* < 0.00001, *I* ^2^ = 95%). Leave-one-out sensitivity analysis demonstrated that no single study disproportionately influenced the pooled effect estimate. Random-effects meta-analysis revealed a notable reduction [n = 1078; MD, −0.87; 95% CI (−1.36, −0.39); *P* = 0.0004] (**Figure 8A**). In general, combined use of GXST capsule on the basis of conventional Western medicine reduced TC levels. According to GRADE evidence quality evaluation, TC was moderate-quality evidence, as shown in **Supplementary File S3**.

##### 3.5.1.2 TG

A total of six studies (16, 22, 27, 31, 32, 36) reported triglyceride (TG) levels, involving a total of 1,078 subjects, including 539 in both the treatment group and the control group. Statistically, there was heterogeneity (*P* < 0.00001, *I* ^2^ = 99%). Pooled analysis using random-effects model indicated measurable changes [n = 1078; MD, −0.62; 95% CI (−1.09, −0.16); *P* = 0.009] (**Figure 8B**), in general, combined use of GXST capsule on the basis of conventional Western medicine reduced TG levels. Sensitivity analysis by leave-one-out method showed that there was still significant heterogeneity in the combined results of the remaining studies; however, when one trial (36) was excluded, there was no significant difference in TG between the two groups [n = 716; MD, −0.52; 95% CI (−1.10, 0.07); *P* = 0.08] (**Figure 8C**), which was attributed to the quality of the included papers. According to GRADE evidence quality evaluation, TG was very low-quality evidence, as shown in **Supplementary File S3**.

##### 3.5.1.3 HDL-C

A total of five studies (16, 27, 31, 32, 36) reported high-density lipoprotein cholesterol (HDL-C) levels, involving a total of 964 subjects, including 482 in both the treatment group and the control group. Statistically, there was heterogeneity (*P* < 0.00001, *I* ^2^ = 97%). Leave-one-out sensitivity analysis demonstrated that no single study disproportionately influenced the pooled effect estimate. Meta-analysis with random-effects model showed positive outcomes [n = 964; MD, 0.40; 95% CI (0.12, 0.68); *P* = 0.005] (**Figure 8D**). In general, combined use of GXST capsule on the basis of conventional Western medicine increased HDL-C levels. According to GRADE evidence quality evaluation, HDL-C was low-quality evidence, as shown in **Supplementary File S3**.

##### 3.5.1.4 LDL-C

A total of five studies (16, 27, 31, 32, 36) reported low-density lipoprotein cholesterol (LDL-C) levels, involving a total of 964 subjects, including 482 in both the treatment group and the control group. Statistically, there was heterogeneity (*P* < 0.00001, *I* ^2^ = 97%). Leave-one-out sensitivity analysis demonstrated that no single study disproportionately influenced the pooled effect estimate. Significant decreases were observed via random-effects model [n = 964; MD, −0.85; 95% CI (−1.16, −0.54); *P* < 0.00001] (**Figure 8E**). In general, combined use of GXST capsule on the basis of conventional Western medicine reduced LDL-C levels. According to GRADE evidence quality evaluation, LDL-C was low-quality evidence, as shown in **Supplementary File S3**.

#### 3.5.2 Inflammatory factors

Pooled analyses of inflammatory factors demonstrated consistent reduction trends with GXST combination therapy, although evidence quality varied (GRADE: low to very low). Key findings include the following.

##### 3.5.2.1 Pro-inflammatory cytokines

Significant decreases in interleukin-6 (IL-6) (SMD, −1.54; 95% CI, −2.23 to −0.85; P < 0.001; n = 716, I² = 93%) and tumor necrosis factor–α (TNF-α) (SMD = −1.68; 95% CI, −2.64 to −0.71; P = 0.001; n = 758, I² = 97%) were observed. Interleukin-1 (IL-1) reduction became non-significant after excluding one outlier study (22; n = 222; SMD, −1.41; 95% CI, −3.04 to 0.22; *P* = 0.09).

##### 3.5.2.2 hs-CRP

**Figure 9:**
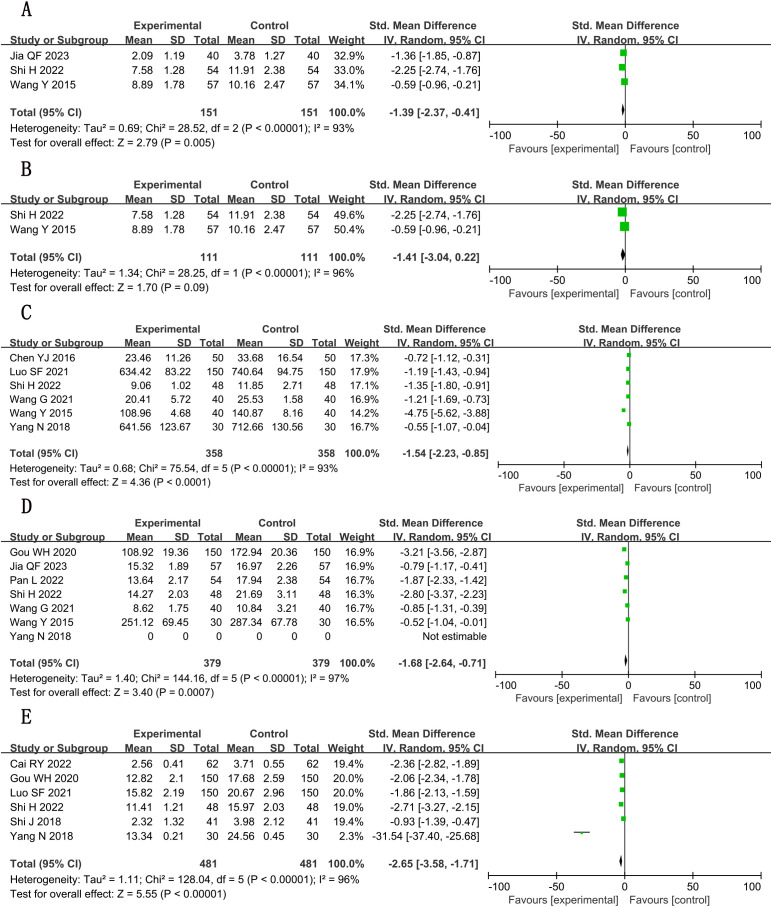
Forest plots for meta-analysis. **(A)** IL-1; **(B)** IL-1 (adjusted); **(C)** IL-6; **(D)** TNF-α; **(E)** hs-CRP.

Marked reduction (SMD, −2.65; 95% CI, −3.58 to −1.71; P < 0.001; n = 962, I² = 96%), although with substantial heterogeneity. Detailed forest plots are presented in **Figures 9A–E**, with full GRADE assessments in **Supplementary File S3**.

#### 3.5.3 Hemorheological parameters

**Figure 10:**
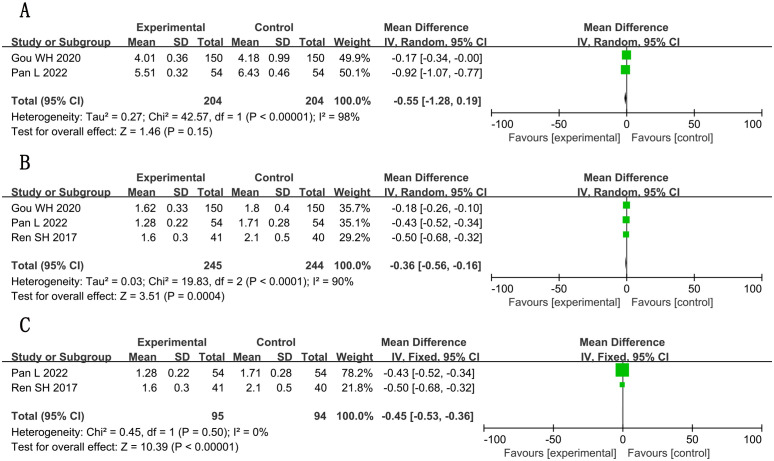
Forest plots for meta-analysis. **(A)** whole-blood viscosity; **(B)** plasma viscosity; **(C)** plasma viscosity (adjusted).

Plasma viscosity showed significant improvement post-sensitivity analysis (MD, −0.45; 95% CI, −0.53 to −0.36; P < 0.001; n = 189, I² = 0%). Whole-blood viscosity changes were non-significant (MD, −0.55; 95% CI, −1.28 to 0.19; P = 0.15; n = 408; I² = 98%). See **Figures 10A–C** for complete analysis. All hemorheology outcomes were graded as low/very low-quality evidence (**Supplementary File S3**).

#### 3.5.4 Cardiac function (LVEF)

**Figure 11:**
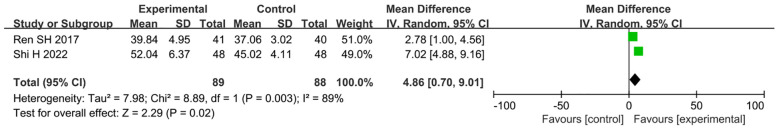
Meta-analysis results of LVEF.

A total of two studies (30, 31) reported LVEF, involving a total of 177 subjects, including 89 in the treatment group and 88 in the control group. Statistically, there was heterogeneity (*P* = 0.003, *I* ^2^ = 89%). Random-effects modeling detected meaningful changes [n = 177; MD, 4.86; 95% CI (0.70, 9.01); *P* = 0.02] (**Figure 11**). In general, combined use of GXST capsule on the basis of conventional Western medicine improved LVEF. According to GRADE evidence quality evaluation, LVEF was low-quality evidence, as shown in **Supplementary File S3**.

### 3.6 Adverse reactions

**Figure 12:**
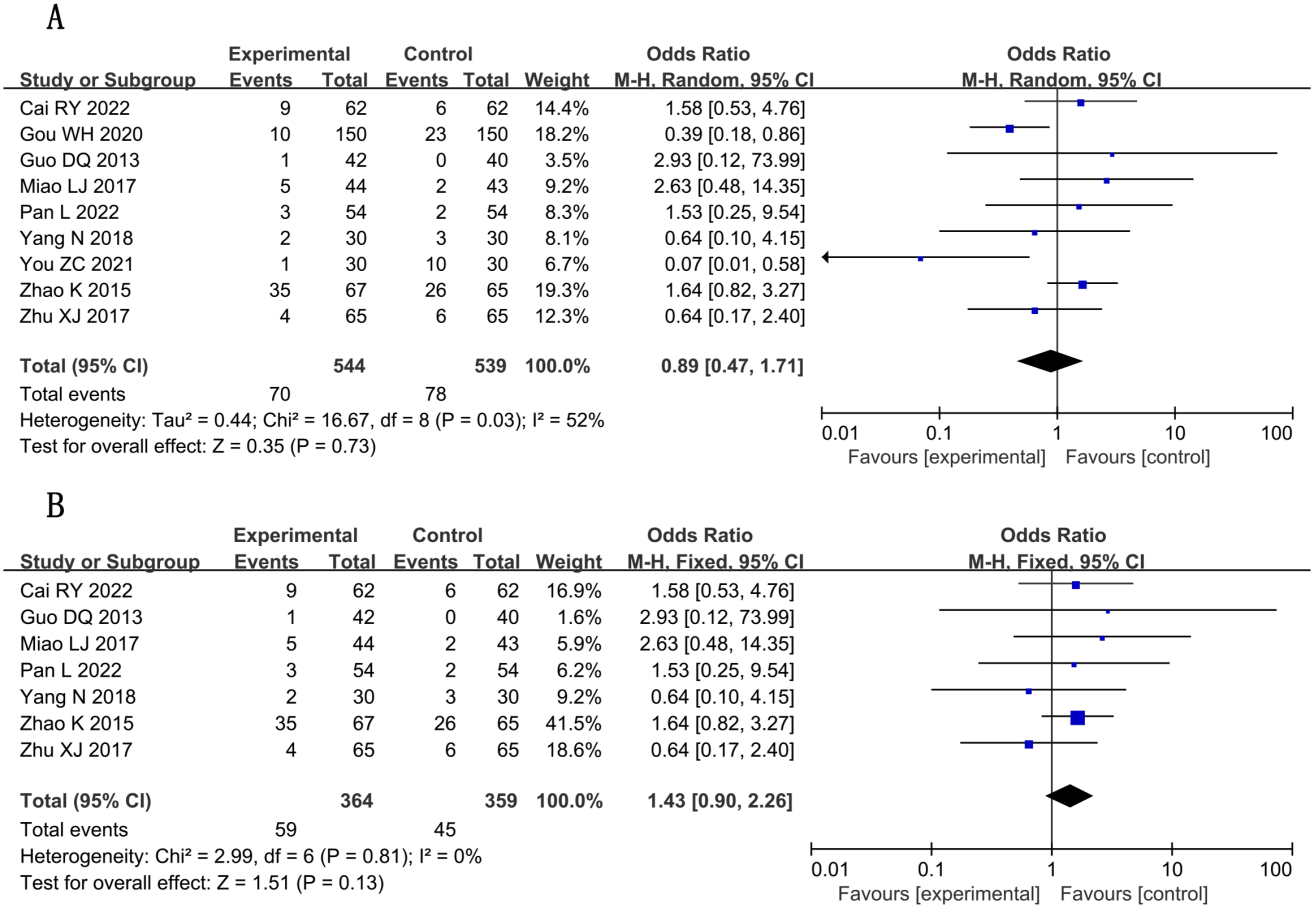
Forest plots for meta-analysis. **(A)** adverse reactions; **(B)** adverse reactions (adjusted).

A total of nine studies (16, 19, 20, 28, 29, 37–39, 41) reported adverse reactions, involving a total of 1,083 subjects, including 544 in the treatment group and 539 in the control group. Statistically, there was heterogeneity (*P* = 0.73, *I* ^2^ = 52) (**Figure 12A**), so sensitivity analysis was performed. By excluding two studies (19, 38), the heterogeneity was reduced to 0%. Fixed-effects analysis post-heterogeneity adjustment revealed patterns [n = 723; MD, 1.43; 95% CI (0.90, 2.26); *P* = 0.13] (**Figure 12B**). In general, combined use of GXST capsule on the basis of conventional Western medicine did not increase adverse reactions. According to GRADE evidence quality evaluation, adverse reactions were low-quality evidence, as shown in **Supplementary File S3**.

### 3.7 Publication bias

**Figure 13:**
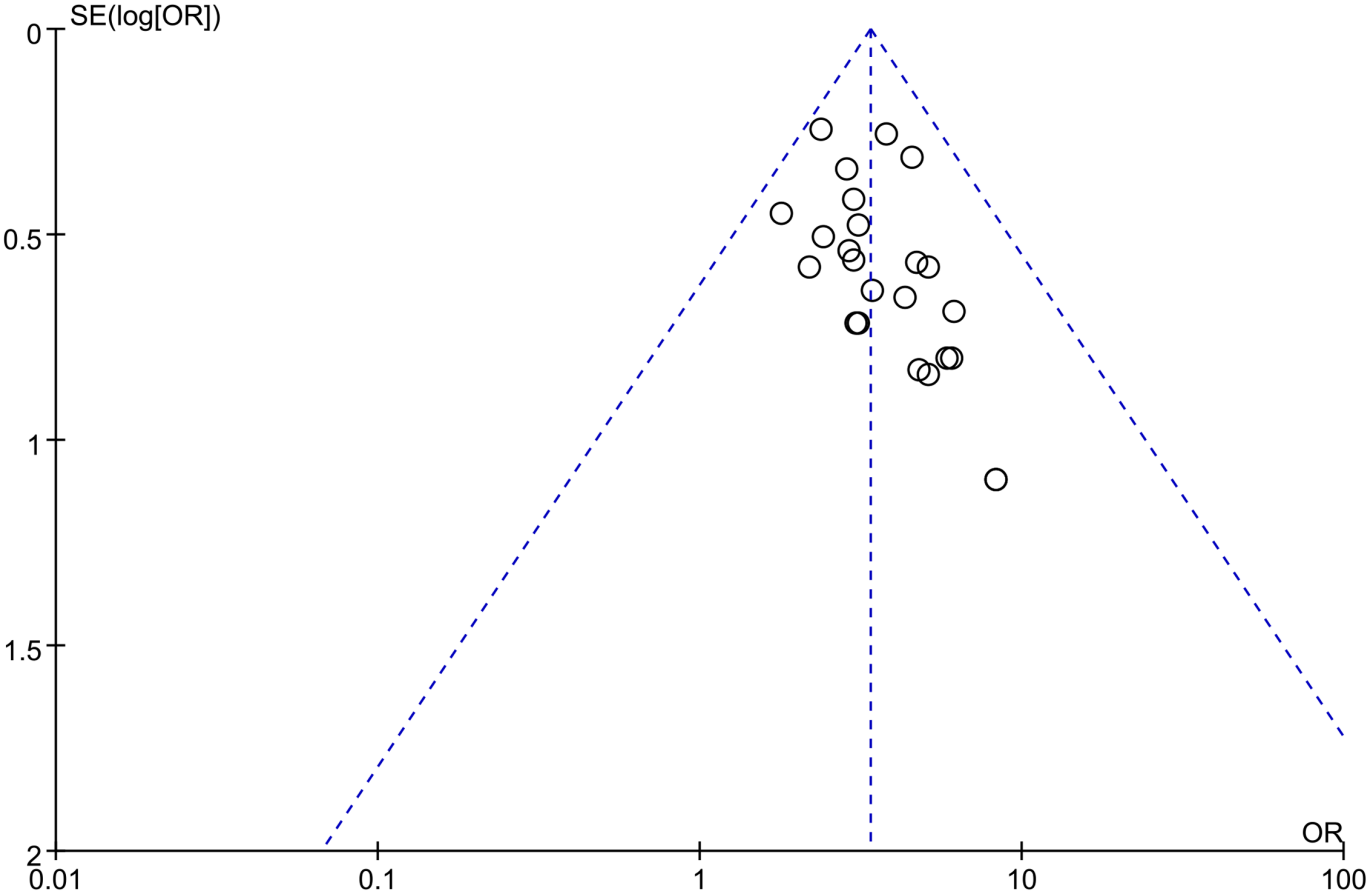
Publication bias graph of the total effective rate of angina pectoris.

Projects with more than 10 outcome indicators were included in this meta-analysis to assess the risk of publication bias. The results showed that the total effective rate of angina pectoris was more evenly distributed on both sides of the midline, indicating a low risk of publication bias (**Figure 13**).

### 3.8 Subgroup analysis

Subgroup analysis was performed on some outcomes based on the two main clinical types of angina pectoris, stable angina pectoris, and unstable angina pectoris. The results showed that there was a subgroup effect on IL-6, although GXST capsule combined with conventional Western medicine could reduce IL-6 levels in both subgroups, the effect was better in the SAP subgroup. In terms of reducing TNF-α and high-sensitivity C-reactive protein (hs-CRP), GXST capsule combined with conventional Western medicine did not show superior efficacy to conventional Western medicine in the UAP subgroup, whereas it did in the SAP subgroup, and there was no subgroup effect on other outcomes, as shown in **Table 2**. The forest chart for each outcome is detailed in **Supplementary File S4**.

**Table 2:** Subgroup analysis on the types of angina pectoris.

Subgroup analysis		Number of studies (patients)	SMD, 95% CI	P overall effect	P interaction
Total effective rate of angina pectoris	SAP	13 (1,988)	1.29 (1.22, 1.35)	<0.00001	0.10
	UAP	6 (704)	1.20 (1.12, 1.28)	<0.00001	
Frequency of angina pectoris	SAP	3 (465)	−1.50 (−2.83, −0.16)	0.03	0.70
	UAP	3 (487)	−1.90 (−3.48, −0.33)	0.02	
Duration of angina pectoris	SAP	2 (178)	−1.27 (−1.92, −0.62)	0.0001	0.86
	UAP	3 (487)	−1.20 (−1.62, −0.97)	<0.00001	
Total effective rate of ECG	SAP	4 (654)	2.25 (1.49, 3.39)	0.0001	0.40
	UAP	3 (249)	1.66 (0.93, 2.95)	0.09	
IL-6	SAP	4 (556)	−2.03 (−3.03, −1.04)	<0.0001	0.01
	UAP	2 (160)	−0.65 (−0.97, −0.34)	<0.0001	
TNF-α	SAP	4 (398)	−1.56 (−2.43, −0.69)	0.0004	0.83
	UAP	2 (360)	−1.87 (−4.51, 0.76)	0.16	
hs-CRP	SAP	3 (478)	−1.82 (−2.66, −0.98)	<0.0001	0.31
	UAP	2 (360)	−16.65 (−45.54, 12.24)	0.26	
Adverse reactions	SAP	4 (657)	1.48 (0.87, 2.53)	0.15	0.32
	UAP	4 (529)	0.82 (0.29, 2.31)	0.70	

SAP, stable angina pectoris; UAP, unstable angina pectoris.

## 4 Discussion

GXST capsule is mainly composed of Fructus choerospondiatis, salvia miltiorrhiza, clove, borneol, and concretio silicea bambusae. Previous studies have shown that GXST capsule mainly has the effects of anti-cardiomyocyte apoptosis, increasing myocardial energy metabolism, regulating blood lipids, inhibiting thrombosis, reducing inflammation levels, anti-oxidative stress, and promoting angiogenesis (42, 43). GXST capsule can delay the progression of atherosclerosis by regulating lipid profile and downregulating the levels of inflammatory cytokines [e.g., TNF-α, IL-1β, IL-6, and Intercellular Cell Adhesion Molecule-1 (ICAM-1)] and Nuclear Factor kappa-light-chain-enhancer of activated B cells (NF-κB) p65 (43, 44). In addition, GXST capsule can improve the left ventricular function of rats after Acute Myocardial Infarction (AMI) surgery by increasing the blood vessel density in the infarction area, improving the myocardial pathological morphology, alleviating fibrosis, reducing the infarct size, and reducing Apoptotic Index (AI). Histologically, GXST capsule can ameliorate myocardial pathologic changes and improve myocardial fibrotic areas and cardiac hypertrophy by upregulating intercellular connexins (N-cad and Cx-43) and downregulating angiogenis-associated proteins [Platelet-Derived Growth Factor (PDGF) and Vascular Endothelial Growth Factor A (VEGFA)], myocardial fibrosis-associated proteins (TGF-β1), and matrix metalloproteinases (MMP-2 and MMP-9) to resist myocardial ischemic pathological changes (45, 46). These results provide a basis for the clinical application of GXST capsule in CHD angina pectoris.

A total of 27 studies were included in this meta-analysis, including 3,440 participants, to investigate the efficacy and safety of GXST capsule combined with conventional Western medicine in the treatment of CHD angina pectoris, as compared with conventional Western medicine alone. The results suggested that the combined use of GXST capsule had better effect on the total effective rate of angina pectoris, frequency of angina pectoris, duration of angina pectoris, and total effective rate of ECG. In reducing TC, LDL-C, IL-6, TNF-α, hs-CRP, and whole-blood viscosity and in increasing LVEF and HDL-C, the combined use of GXST capsule had more advantages. However, for TG and IL-1, there were two different results before and after the sensitivity analysis, which were attributed to the quality of the included literature. In terms of plasma viscosity and adverse reactions, after excluding the literature with large heterogeneity, sensitivity analysis indicated that the combined use of GXST capsule was helpful to reduce plasma viscosity and adverse reactions. Overall, the systematic review provided evidence that combined use of GXST capsule on the basis of conventional Western medicine was more beneficial than Western medicine alone.

Although there have been three systematic reviews on the treatment of angina pectoris of CHD with GXST capsule on CNKI (47–49), our study provides additional perspectives. The main differences between our study and previous studies are as follows: firstly, more literatures were included in our study. The most recent systematic review has been published for over 5 years (47), during which time many new findings, which were not systematically investigated, were supplemented by our study. Secondly, compared to previous studies, inflammatory factors such as IL-6, IL-1, and TNF-α, as well as hemorheological and cardiac function indicators, which are closely related to the occurrence of CHD and cardiovascular adverse events and have not been studied previously, were added in our study. Thirdly, previous studies have not evaluated the quality of outcomes, whereas the GRADE evidence quality rating was added in our study to rate the quality of evidence for the included outcome indicators, clearly demonstrating the quality of evidence and strength of recommendation for each outcome. Our study has certain advantages. Previous studies have only evaluated the efficacy of GXST capsule in stable angina pectoris (47) or unstable angina pectoris (48, 49), whereas our study included all types of angina pectoris and evaluated the overall efficacy of GXST capsule in treating angina. The quality of our study was controlled more strictly, the RCTs with a sample size of 60 or more were selected, and stricter inclusion and exclusion criteria were adopted to improve the quality of evidence and reduce the risk of bias. Finally, subgroup analyses were added in our study to determine the efficacy and safety of GXST capsule under specific circumstances.

Systematic review and meta-analysis in this study were conducted in strict accordance with the PRISMA guidelines to evaluate the degree of standardization of clinical trials from a methodological perspective. However, our study still has certain limitations: some original studies were of low quality, with mostly uncertain risks in terms of allocation concealment and blinding of investigators and data processing; The number of literatures on some indicators was small, thus not allowing for bias detection. The sample sizes of some studies were still small.

## 5 Conclusions

In summary, GXST capsule combined with conventional Western medicine has superior efficacy and safety to Western medicine alone in the treatment of CHD angina pectoris. Our study provides a direction for further research and a guidance for future clinical studies to be more standardized and rigorous in design. In future studies, more standardized RCTs are needed to verify the above conclusions, and longer follow-up periods shall be designed to explore its long-term efficacy.

## Data Availability

The datasets presented in this study can be found in online repositories. The names of the repository/repositories and accession number(s) can be found in the article/**Supplementary Material**.

## References

[1] Krievins DK Zellans E Latkovskis G Kumsars I Krievina AK Jegere S . Diagnosis and treatment of ischemia-producing coronary stenoses improves 5-year survival of patients undergoing major vascular surgery. J Vasc Surg. (2024) S0741-5214:00500–7. doi: 10.1016/j.jvs.2024.02.043 38518962

[2] Al-Lamee RK . Angina pectoris 2023: With and without obstructive coronary artery disease: Epidemiology, diagnosis, prognosis, and treatment. Vasc Pharmacol. (2024) 155:107285. doi: 10.1016/j.vph.2024.107285 38431201

[3] Balla C Pavasini R Ferrari R . Treatment of angina: where are we? Cardiology. (2018) 140:52–67. doi: 10.1159/000487936 29874661

[4] Henry TD Satran D Jolicoeur EM . Treatment of refractory angina in patients not suitable for revascularization. Nat Rev Cardiol. (2014) 11:78–95. doi: 10.1038/nrcardio.2013.200 24366073

[5] Münzel T Daiber A Gori T . More answers to the still unresolved question of nitrate tolerance. Eur Heart J. (2013) 34:2666–73. doi: 10.1093/eurheartj/eht249 23864131

[6] Jia Y Gao G Leung SW . How efficacious are traditional Chinese medicine injections in treating angina pectoris? A network meta-analysis of randomized controlled trials. J Ethnopharmacol. (2023) 303:115996. doi: 10.1016/j.jep.2022.115996 36509258

[7] Luo J Xu H Yang G Qiu Y Liu J Chen K . Oral Chinese proprietary medicine for angina pectoris: an overview of systematic reviews/meta-analyses. Complement. Ther Med. (2014) 22:787–800. doi: 10.1016/j.ctim.2014.05.011 25146083

[8] Cui X Han S Li J Li W Wang ZF Zhang Q . Clinical comprehensive evaluation of Guanxin Shutong Capsules in treatment of coronary heart disease angina pectoris with heart blood stasis syndrome. China J Chin. Mater Med. (2022) 47:1469–75. doi: 10.19540/j.cnki.cjcmm.20211118.501 35347945

[9] Tian S Wang JM Li YY Li D Xu L Hou TJ . The application of in silico drug-likeness predictions in pharmaceutical research. Adv Drug Deliv. Rev. (2015) 86:2–10. doi: 10.1016/j.addr.2015.01.009 25666163

[10] Knobloch K Yoon U Vogt PM . Preferred reporting items for systematic reviews and meta-analyses (PRISMA) statement and publication bias. J Craniomaxillofac. Surg. (2011) 39:91–2. doi: 10.1016/j.jcms.2010.11.001 21145753

[11] Liberati A Altman DG Tetzlaff J Mulrow C Gøtzsche PC Ioannidis JP . The PRISMA statement for reporting systematic reviews and meta-analyses of studies that evaluate health care interventions: explanation and elaboration. Ann Intern Med. (2009) 151:W65–94. doi: 10.7326/0003-4819-151-4-200908180-00136 19622512

[12] Zhang X Geng P Zhang TT Lu Q Gao P Mei J . Aceso: PICO-guided evidence summarization on medical literature. IEEE J Biomed Health Inform. (2020) PP:(3). doi: 10.1109/JBHI.2020.2984704 32275627

[13] McKenzie JE Brennan SE Ryan RE Thomson HJ Johnston RV . Chapter9: summarizing study characteristics and preparing for synthesis. In: Higgins JPT Thomas j. Chandler J Cumpston M Li T Page MJ Welch VA , editors. Cochrane Handbook for Systematic Reviews of Interventions Version 6.3 (updated February 2022). London: Cochrane (2022). Available at: www.training.cochrane.org/handbook (Accessed January 21, 2024).

[14] Sterne JAC Savovié J Page MJ Elbers RG Blencowe NS Boutron I . RoB 2: a revised tool for assessing risk of bias in randomised trials. BMJ. (2019) 366:14898. doi: 10.1136/bmj.148983 31462531

[15] Li Y Zhang L Lv SZ Wang XZ Zhang J Tian XX . Efficacy and safety of oral Guanxinshutong capsules in patients with stable angina pectoris in China: a prospective, multicenter, double-blind, placebo-controlled, randomized clinical trial. BMC Complement Altern. Med. (2019) 19:363. doi: 10.1186/s12906-019-2778-z 31829173 PMC6907120

[16] Cai RY . Treatment effects, safety and effective rates of guanxin shutong capsule in angina pectoris with hypertension. Liaoning J Tradit. Chin. Med. (2022) 49:127–30. doi: 10.13192/j.issn.1000-1719.2022.06.035

[17] Cao XY Yue YY . Clinical effect of Guanxin Shutong capsule on angina pectoris of coronary heart disease. Guid. Chin. Med. (2013) 11:249–50. doi: 10.15912/j.cnki.gocm.2013.26.030

[18] Chen YJ Chen YQ Xiao Y Liu D Cao XH Zhao HM . The influence of guanxin shutong capsule and trimetazidine dihydrochloride on the expressions of IL-6 and NT-proBNP in patients with unstable angina pectoris. Chin. J Integr Med Cardio-Cerebrovasc. Dis. (2016) 14:2484–6. doi: 10.3969/j.issn.1672-1349.2016.21.005

[19] Gou WH Zhang D . Efficacy of guanxinshutong capsule combined with metoprolol in the treatment of unstable angina pectoris and its effects on hemorheology and inflammatory factors. Eval. Anal Drug-Use Hosp. China. (2020) 20:1075–1077 + 1082. doi: 10.14009/j.issn.1672-2124.2020.09.013

[20] Guo DQ Li P . Clinical observation of Guanxinshutong capsule in the treatment of unstable angina pectoris. Chin. J Clin. (2013) 41:37–8. doi: 10.3969/j.issn.1008-1089.2013.12.013

[21] Ji YY . Effect of Guanxinshutong capsule on stable angina pectoris of coronary heart disease. Cardiovasc Dis J Integr Tradit. Chin. West Med. (2017) 5:169 + 172. doi: 10.16282/j.cnki.cn11-9336/r.2017.01.127

[22] Jia QF Li XW . Clinical observation of Guanxinshutong capsule combined with fluvastatin in the treatment of angina pectoris of coronary heart disease. J Pract Tradit. Chin. Med. (2023) 39:1555–7. Available at: https://www.cnki.com.cn/Article/CJFDTOTAL-ZYAO202308028.htm (Accessed December 21, 2023).

[23] Li XB Li JX Li ZH . Effect of Guanxinshutong capsule on myocardial insufficiency of coronary heart disease. Pract J Card Cereb Pneumal Vasc Dis. (2014) 22:131–2. Available at: https://www.cnki.com.cn/Article/CJFDTOTAL-SYXL201409078.htm (Accessed December 21, 2023).

[24] Liang B . Clinical observation of 43 cases of unstable angina pectoris treated with Guanxinshutong capsule. Yunnan J Tradit. Chin. Med Mater Med. (2015)36:23–4. doi: 10.16254/j.cnki.53-1120/r.2015.02.011

[25] Lin YC . Effect of Guanxinshutong capsule on vascular endothelial cell function in patients with unstable angina pectoris. J New Chin. Med. (2016) 48:18–20. doi: 10.13457/j.cnki.jncm.2016.05.008

[26] Liu SS Li J Shi YX Huang ZZ Li ZG . Effect of Guanxinshutong capsule combined with metoprolol on angina pectoris and plasma homocysteine level in elderly patients with coronary heart disease. Mod J Integr Tradit. Chin. West Med. (2019) 28:1892–5. doi: 10.3969/j.issn.1008-8849.2019.17.017

[27] Luo SF . The Clinical Efficacy of Coronary Heart Sultan Capsule combined with Western Medicine to Treat Coronary Heart Disease Stable Angina and its Effect on Blood Lipids and hs-CRP. Smart Healthc. (2021) 7:173–5. doi: 10.19335/j.cnki.2096-1219.2021.08.056

[28] Miao LJ Cheng GS . Effect of Guanxinshutong Capsule on electrocardiogram and myocardial enzyme in patients with unstable angina pectoris. Asia-Pac. Tradit. Med. (2017) 13:152–4. Available at: https://www.cnki.com.cn/Article/CJFDTOTAL-YTCT201716065.htm (Accessed December 21, 2023).

[29] Pan L Li P Ouyang Sk Song H Li JX . Effects of guanxinshutong capsule combined with nicorandil on cardiac function, hemorheology and inflammatory factors in patients with stable angina pectoris and heart blood stasis type. Prog Mod Biomed. (2022) 22:4125–4129 + 4152. doi: 10.13241/j.cnki.pmb.2022.21.022

[30] Ren SH Zhang RJ Ke SX Liu JB Shi YF Guo Y . Effects of Guanxinshutong Capsule on cardiac function and hemorheology in patients with coronary heart disease. J Pract Tradit. Chin. Med. (2017) 33:492–3. Available at: https://www.cnki.com.cn/Article/CJFDTOTAL-ZYAO201705022.htm (Accessed December 21, 2023).

[31] Shi H . Effect of guanxin shutong capsule(冠心舒通胶囊) combined with metoprolol in treatment of stable angina pectoris of coronary heart disease and its influence on blood lipids and hs-CRP. Liaoning J Tradit. Chin. Med. (2022) 49:137–40. doi: 10.13192/j.issn.1000-1719.2022.09.039

[32] Shi J Abudujili A . Clinical study of Guanxinshutong capsule in treating stable angina pectoris of coronary heart disease. Chin. J Integr Med Cardio-Cerebrovasc. Dis. (2018) 16:199–201. Available at: https://www.cnki.com.cn/Article/CJFDTOTAL-ZYYY201802020.htm (Accessed December 21, 2023).

[33] Song JH . Exploring the effect of Guanxin Shutong capsule combined with Western medicine in the treatment of coronary heart disease angina pectoris. Cardiovasc Dis J Integr Tradit. Chin. West Med. (2016) 4:39 + 41. doi: 10.16282/j.cnki.cn11-9336/r.2016.04.022

[34] Wang G . Clinical effect of Guanxinshutong capsule combined with isosorbide mononitrate tablet on patients with angina pectoris of coronary heart disease. Med Forum. (2021) 25:648–50. doi: 10.19435/j.1672-1721.2021.05.028

[35] Wang Y Wu JS . Observe the clinical effect of Guanxin Shutong capsule treatment of Coronary heart disease with cariac blood stasis syndrome. Clin J Tradit. Chin. Med. (2015) 27:666–8. doi: 10.16448/j.cjtcm.2015.0253

[36] Wu ZJ Huang XX Chen J . Clinical observation of Guanxinshutong capsule combined with Western medicine in the treatment of angina pectoris of coronary heart disease. J New Chin. Med. (2015) 47:43–4. doi: 10.13457/j.cnki.jncm.2015.01.019

[37] Yang N Zhang JJ . Clinical effect of Guanxin Shutong capsule combined with conventional western medicine on angina pectoris and its effect on inflammatory factors. Clin Res Prac. (2018) 3:4243. doi: 10.19347/j.cnki.2096-1413.201828018

[38] You ZC . Clinical efficacy and safety analysis of patients with coronary heart disease in patients with coronary heart disease in patients with coronary heart disease. Syst Med. (2021) 6:88–90. doi: 10.19368/j.cnki.2096-1782.2021.19.088

[39] Zhao JS Zhang YQ . Effects of Guanxin-shutong capsule on QT dispersion in patients with unstable angina pectoris. Hebei J Tradit. Chin. Med. (2015) 37:1698–700. doi: 10.3969/j.issn.1002-2619.2015.11.035

[40] Zhao K Meng K Ge CJ Lv SZ . Curative effect of Guanxin Shutong Capsules on psychocardiacology in patients with chronic stable angina pectoris (syndrome of heart blood stasis). Chin. J Evid. Based Cardiovasc Med. (2015) 7:184–7. doi: 10.3969/j.1674-4055.2015.02.10

[41] Zhu XJ Liu J . Effect of Guanxinshutong capsule on patients with angina pectoris complicated with hypertension. Hebei Med J. (2017) 39:269–71. doi: 10.3969/j.issn.1002-7386.2017.02.033

[42] Liang Z Liu LF Yao TM Huo Y Han YL . Cardioprotective effects of Guanxinshutong (GXST) against myocardial ischemia/reperfusion injury in rats. J Geriatr. Cardiol. (2012) 9:130–6. doi: 10.3724/SP.J.1263.2011.11261 PMC3418901 22916058

[43] Lu YD Sun YC Jiang ZL Zhang DD Lin HC Qu Y . Guanxinshutong alleviates atherosclerosis by suppressing oxidative stress and proinflammation in apoE-/- mice. Evid. Based Complement Alternat. Med. (2020) 2020:1219371. doi: 10.1155/2020/1219371 PMC7519182 33014098

[44] Liang Z Yao TM Huo Y Han YL . The effects of Guanxinshutong on protection of left ventricular function after acute myocardial infarction in rats. Chin. J Intern Med. (2012) 51:225–7. doi: 10.3760/cma.j.issn.0578-1426.2012.03.013 22781899

[45] Jiang LH Wu CZ . Effeets of myocardial angiogenesis on Guanxin Shutong capsules in acute myocardial infaretion rats. J Changchun Univ Chin. Med. (2015) 31:451–3. doi: 10.13463/j.cnki.cczyy.2015.03.005

[46] Zhu JQ Zhou HF Li C He Y Pan YM Shou QY . Guanxinshutong capsule ameliorates cardiac function and architecture following myocardial injury by modulating ventricular remodeling in rats. BioMed Pharmacother. (2020) 130:110527. doi: 10.1016/j.biopha.2020.110527 32688142

[47] Xi YT Wang SD Yuan LY Liu XY Wu W . Guanxin shutong capsule in the adjuvant treatment of unstable angina pectoris: A meta-analysis and trial sequential analysis. China Pharm. (2019) 30:956–62. doi: 10.6039/j.issn.1001-0408.2019.07.20 31602958

[48] Jia WX Wei FX . Meta analysis of clinical effects and the safety of guanXin shuTong capsules in treating stable angina pectoris. West. J Tradit. Chin. Med. (2017) 30:67–70. Available at: https://www.cnki.com.cn/Article/CJFDTOTAL-GSZY201708021.htm (Accessed December 21, 2023).

[49] Sui JY Xu H Qin L Xu GL . A meta-analysis of guanxin shutong capsule on patients with unstable angina. J Emerg Tradit. Chin. Med. (2016) 25:1863–1865 + 1893. doi: 10.3969/j.issn.1004-745X.2016.10.009

