# Supplementary material for: Meta-analysis on the efficacy and safety of Guanxin Shutong capsule in the treatment of angina pectoris of coronary heart disease
Source: Front Endocrinol (Lausanne). 2025 Jun 17;16:1534752. doi: 10.3389/fendo.2025.1534752 (PMC12208842; doi:10.3389/fendo.2025.1534752)
Supplement: Supplementary file 1 [file DataSheet1.docx]

**Supplementary File S1. （table of the main botanical drug metabolites of GXST capsule）**

| **English/Latin Name** | **Chinese Name** | **Family** | **Species** | **Molecule ID** | **Molecule** |
| --- | --- | --- | --- | --- | --- |
| Choerospondiatis fructus/Choerospondias axillaris (Roxb.) B.L.Burtt & A.W.Hill | Guangzao | Anacardiaceae | Choerospondias axillaris | MOL001040 | (2R)-5,7-dihydroxy-2-(4- hydroxyphenyl)chroman-4-one |
|  |  |  |  | MOL001736 | (-)-taxifolin |
|  |  |  |  | MOL000358 | beta-sitosterol |
|  |  |  |  | MOL000422 | kaempferol |
|  |  |  |  | MOL004328 | naringenin |
|  |  |  |  | MOL000098 | quercetin |
| Salviae miltiorrhizae radix et rhizoma / Salvia miltiorrhiza Bunge | Danshen | Lamiaceae | S. miltiorrhiza | MOL001601 | 1,2,5,6-tetrahydrotanshinone |
|  |  |  |  | MOL001659 | Poriferasterol |
|  |  |  |  | MOL001771 | poriferast-5-en-3beta-ol |
|  |  |  |  | MOL002222 | sugiol |
|  |  |  |  | MOL002651 | Dehydrotanshinone Ⅱ A |
|  |  |  |  | MOL000006 | luteolin |
|  |  |  |  | MOL007036 | 5,6-dihydroxy-7-isopropyl-1,1-  dimethyl-2,3-dihydrophenanthren-4-one |
|  |  |  |  | MOL007041 | 2-isopropyl-8-methylphenanthrene-3,4-dione |
|  |  |  |  | MOL007045 | 3a-hydroxytanshinone ll a |
|  |  |  |  | MOL007048 | (E)-3-[2-(3,4-dihydroxyphenyl)-7-hydroxy-benzofuran-4-yl]acrylic acid |
|  |  |  |  | MOL007049 | 4-methylenemiltirone |
|  |  |  |  | MOL007050 | 2-(4-hydroxy-3-methoxyphenyl)-5-(3-hydroxypropyl)-7-methoxy-3-benzofurancarboxaldehyde |
|  |  |  |  | MOL007058 | formyltanshinone |
|  |  |  |  | MOL007059 | 3-beta-Hydroxymethyllenetanshiquinone |
|  |  |  |  | MOL007061 | Methylenetanshinquinone |
|  |  |  |  | MOL007068 | Przewaquinone B |
|  |  |  |  | MOL007069 | przewaquinone c |
|  |  |  |  | MOL007070 | (6S,7R)-6,7-dihydroxy-1,6-  dimethyl-8,9-dihydro-7H-  naphtho[8,7-g]benzofuran-10,11-  dione |
|  |  |  |  | MOL007071 | przewaquinone f |
|  |  |  |  | MOL007077 | sclareol |
|  |  |  |  | MOL007079 | tanshinaldehyde |
|  |  |  |  | MOL007081 | Danshenol B |
|  |  |  |  | MOL007082 | Danshenol A |
|  |  |  |  | MOL007085 | Salvilenone |
|  |  |  |  | MOL007088 | cryptotanshinone |
|  |  |  |  | MOL007093 | dan-shexinkum d |
|  |  |  |  | MOL007094 | danshenspiroketallactone |
|  |  |  |  | MOL007098 | deoxyneocryptotanshinone |
|  |  |  |  | MOL007100 | dihydrotanshinlactone |
|  |  |  |  | MOL007101 | dihydrotanshinone l |
|  |  |  |  | MOL007108 | isocryptotanshi-none |
|  |  |  |  | MOL007111 | lsotanshinone Ⅱ |
|  |  |  |  | MOL007115 | manool |
|  |  |  |  | MOL007118 | microstegiol |
|  |  |  |  | MOL007119 | miltionone I |
|  |  |  |  | MOL007122 | Miltirone |
|  |  |  |  | MOL007124 | neocryptotanshinone ii |
|  |  |  |  | MOL007125 | neocryptotanshinone |
|  |  |  |  | MOL007127 | 1-methyl-8,9-dihydro-7H-  naphtho[5,6-g]benzofuran-  6,10,11-trione |
|  |  |  |  | MOL007130 | prolithospermic acid |
|  |  |  |  | MOL007140 | (Z)-3-[2-[(E)-2-(3,4-  dihydroxyphenyl)vinyl]-3,4-  dihydroxy-phenyl]acrylic acid |
|  |  |  |  | MOL007149 | NSC 122421 |
|  |  |  |  | MOL007150 | (6S)-6-hydroxy-1-methyl-6-  methylol-8,9-dihydro-7H-  naphtho[8,7-g]benzofuran-10,11-quinone |
|  |  |  |  | MOL007151 | Tanshindiol B |
|  |  |  |  | MOL007152 | Przewaquinone E |
|  |  |  |  | MOL007154 | tanshinone iia |
|  |  |  |  | MOL007155 | (6S)-6-(hydroxymethyl)-1,6-  dimethyl-8,9-dihydro-7H-  naphtho[8,7-g]benzofuran-10,11-dione |
|  |  |  |  | MOL007156 | tanshinone VI |
| English/Latin Name | Chinese Name | Family | Species | Molecule ID | Molecule |
| Eugenia caryophyllata/Syzygium aromaticum (L.) Merr. & L.M.Perry | Dingxiang | Myrtaceae | Syzygium aromaticum | MOL000098 | quercetin |
|  |  |  |  | MOL013219 | Strictosamide_qt |
|  |  |  |  | MOL000358 | beta-sitosterol |
|  |  |  |  | MOL000422 | kaempferol |
|  |  |  |  | MOL000449 | Stigmasterol |
| Cinnamonum camphora/Camphora officinarum Nees | Bingpian | Lauraceae |  | MOL006861 | asiatic acid |
|  |  |  |  | MOL006862 | bronyl acetate |
|  |  |  |  | MOL006865 | dipterocarpol |
| Bambusae concretio silicea/Bambusa textilis McClure | Tianzhuhuang | Poaceae | Bambusa textilis | MOL000041 | PHA |
|  |  |  |  | MOL000042 | LPG |
|  |  |  |  | MOL004743 | Z-Leu-OH |
|  |  |  |  | MOL005449 | h-Met-h |
|  |  |  |  | MOL000061 | Prolinum |
|  |  |  |  | MOL000067 | L-Valin |
|  |  |  |  | MOL000068 | L-lle |

**Note:** The complex compounds of 13 botanical drugs of NXT were obtained from TCMSP (https://www.tcmsp-e.com/#/database), which is the largest noncommercial TCM database worldwide. TCMSP have collected all the 499 herbs registered in Chinese pharmacopoeia (2010), with a total of 12144 chemical（<https://tcmspe.com/load_intro.php?id=40>）

**Supplementary File S2. （The risk assessment of subjective outcomes and objective outcomes）**


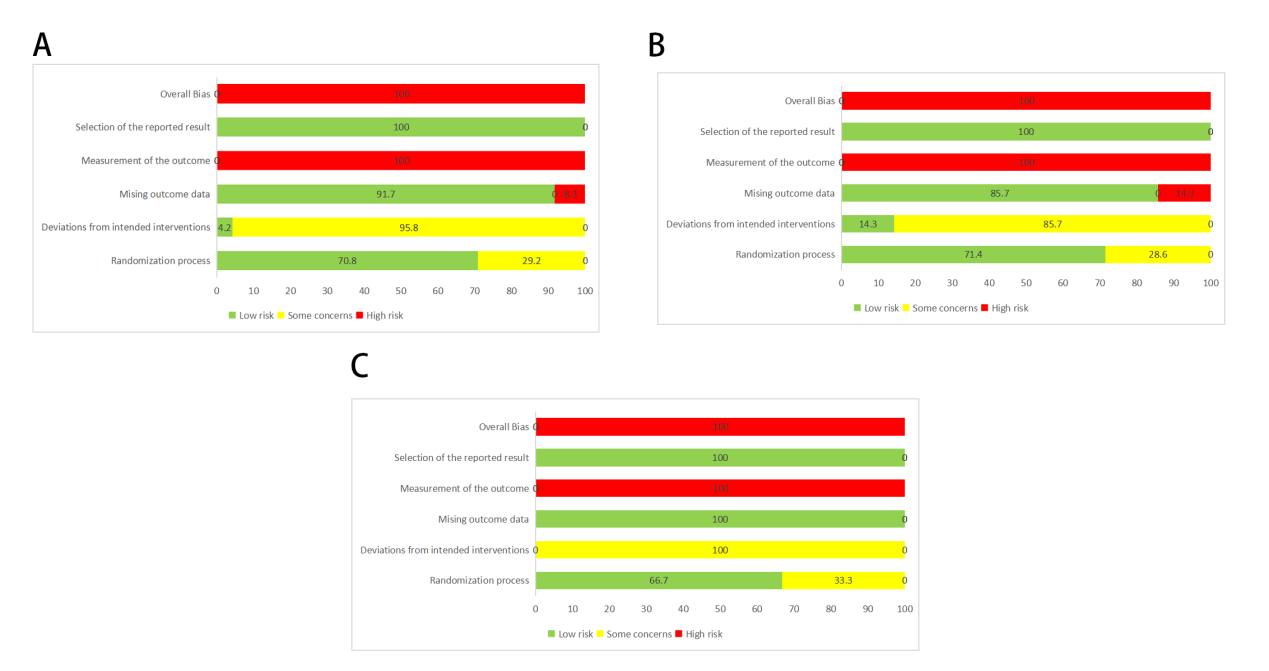


FIGURE 1 The risk assessment of subjective outcomes. (A) Total effective rate of angina pectoris; (B) Frequency of angina pectoris; (C) Duration of angina pectoris


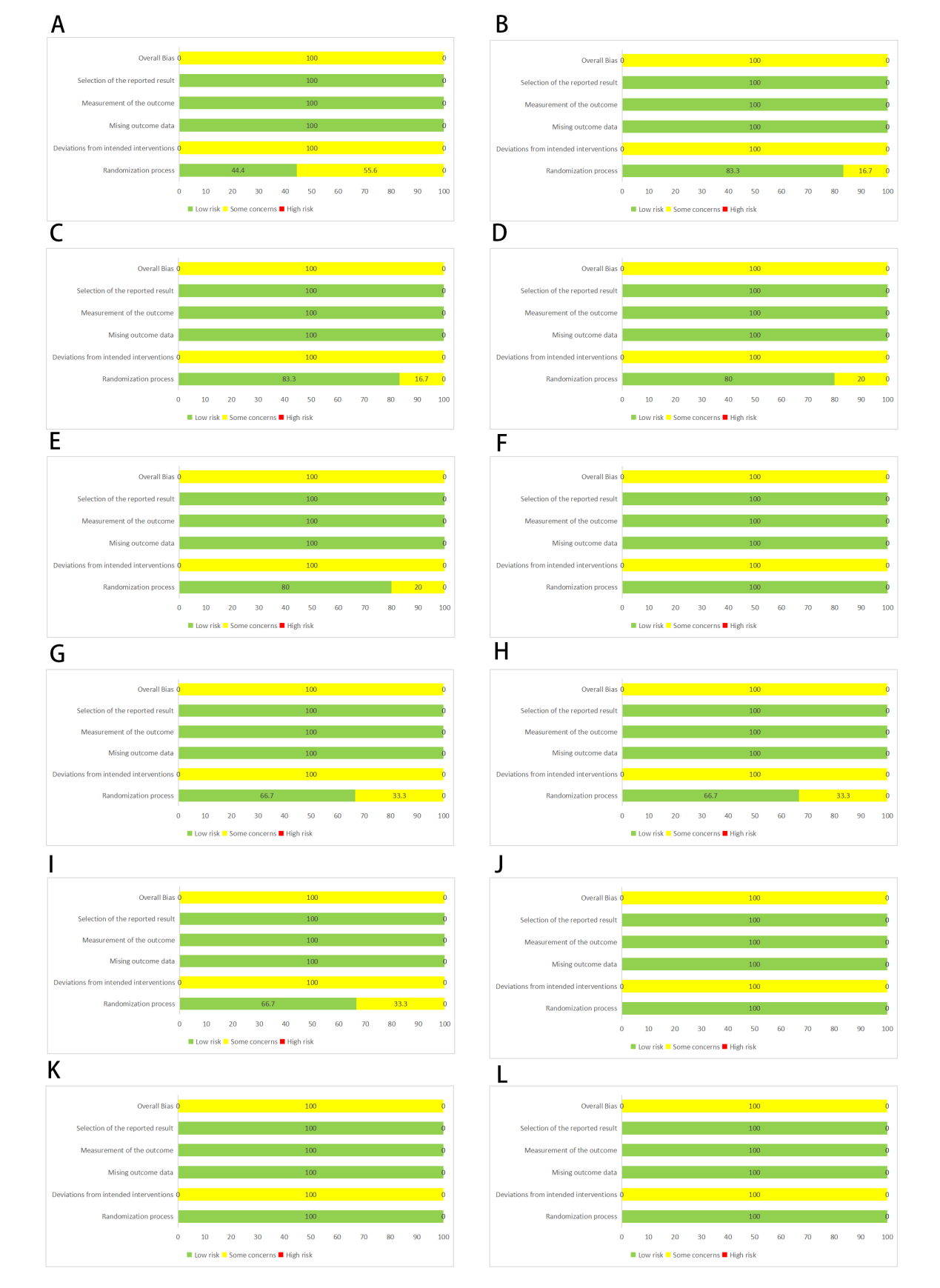


FIGURE 2 The risk assessment of objective outcomes. (A) Total effective rate of ECG; (B) TC; (C) TG; (D) HDL-C; (E) LDL-C; (F) IL-1; (G) IL-6; (H) TNF-α; (I) hs-CRP; (J) Whole blood viscosity; (K) Plasma viscosity; (L) LVEF


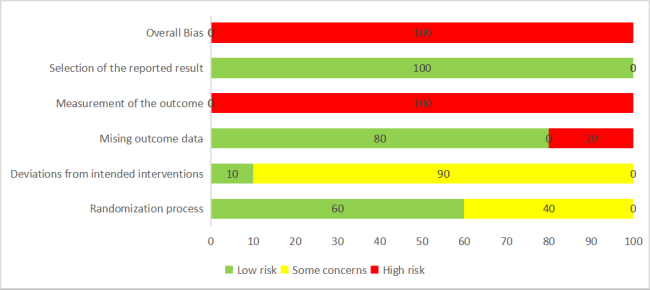


FIGURE 3 The risk assessment of Adverse reactions.

**Supplementary File S3. （Summary of GRADE evidence quality evaluation）**

| Interventions for[Condition] in[Population] | | | | | | | |
| --- | --- | --- | --- | --- | --- | --- | --- |
| **Outcomes** | **Intervention and Comparison intervention** | **Illustrative comparative risks* (95% CI)** | | **Relative effect**  **(95% CI)** | **No. of Participants**  **(studies)** | **Quality of the evidence**  **(GRADE)** | **Comments** |
|  |  | **Assumed risk** | **Corresponding risk** |  |  |  |  |
|  |  | **With comparator** | **With intervention** |  |  |  |  |
| **The total effective rate of angina pectoris** | | | | | | | |
|  | GXST capsule Combined with Conventional Western Medicine/Conventional Western Medicine | **Study population** | | **OR 3.36**  (2.78 to 4.07) | 3187  (24 studies) | ⊕⊕⊕⊝  **moderate** |  |
|  |  | **700 per 1000** | **887 per 1000**  (867 to 905) |  |  |  |  |
|  |  | **Moderate** | |  |  |  |  |
|  |  | **746 per 1000** | **908 per 1000**  (891 to 923) |  |  |  |  |
| **The total effective rate of electrocardiogram** | | | | | | | |
|  | GXST capsule Combined with Conventional Western Medicine/Conventional Western Medicine | **Study population** | | **OR 2.22**  (1.63 to 3.01) | 1068  (9 studies) | ⊕⊕⊕⊝  **moderate** |  |
|  |  | **722 per 1000** | **852 per 1000**  (809 to 886) |  |  |  |  |
|  |  | **Moderate** | |  |  |  |  |
|  |  | **700 per 1000** | **838 per 1000**  (792 to 875) |  |  |  |  |
| **The frequency of angina pectoris** | | | | | | | |
|  | GXST capsule Combined with Conventional Western Medicine/Conventional Western Medicine |  | The mean the frequency of angina pectoris in the intervention groups was  **2.06 standard deviations lower**  (2.22 to 1.9 lower) |  | 1076  (7 studies) | ⊕⊕⊕⊝  **moderate** | SMD -2.06 (-2.22 to -1.9) |
| **The duration of angina pectoris** | | | | | | | |
|  | GXST capsule Combined with Conventional Western Medicine/Conventional Western Medicine |  | The mean the duration of angina pectoris in the intervention groups was  **1.43 standard deviations lower**  (1.58 to 1.27 lower) |  | 789  (6 studies) | ⊕⊕⊝⊝  **low** | SMD -1.43 (-1.58 to -1.27) |
| **TC** | | | | | | | |
|  | GXST capsule Combined with Conventional Western Medicine/Conventional Western Medicine |  | The mean TC in the intervention groups was  **0.87 lower**  (1.36 to 0.39 lower) |  | 1078  (6 studies) | ⊕⊕⊕⊝  **moderate** |  |
| **TG** | | | | | | | |
|  | GXST capsule Combined with Conventional Western Medicine/Conventional Western Medicine |  | The mean TG in the intervention groups was **0.62 lower** (1.09 to 0.16 lower) |  | 1078  (6 studies) | ⊕⊝⊝⊝  **very low** |  |
| **LDL-C** | | | | | | | |
|  | GXST capsule Combined with Conventional Western Medicine/Conventional Western Medicine |  | The mean LDL-C in the intervention groups was  **0.85 lower**  (1.16 to 0.54 lower) |  | 964  (5 studies) | ⊕⊕⊝⊝  **low** |  |
| **HDL-C** | | | | | | | |
|  | GXST capsule Combined with Conventional Western Medicine/Conventional Western Medicine |  | The mean HDL-C in the intervention groups was  **0.4 higher**  (0.12 to 0.68 higher) |  | 964  (5 studies) | ⊕⊕⊝⊝  **low** |  |
| **hs-CRP** | | | | | | | |
|  | GXST capsule Combined with Conventional Western Medicine/Conventional Western Medicine |  | The mean hs-CRP in the intervention groups was  **2.65 standard deviations lower**  (3.58 to 1.71 lower) |  | 962  (6 studies) | ⊕⊕⊝⊝  **low** | SMD -2.65 (-3.58 to -1.71) |
| **IL-6** | | | | | | | |
|  | GXST capsule Combined with Conventional Western Medicine/Conventional Western Medicine |  | The mean IL-6 in the intervention groups was  **1.54 standard deviations lower**  (2.23 to 0.85 lower) |  | 716  (6 studies) | ⊕⊕⊝⊝  **low** | SMD -1.54 (-2.23 to -0.85) |
| **TNF-α** | | | | | | | |
|  | GXST capsule Combined with Conventional Western Medicine/Conventional Western Medicine |  | The mean TNF-α in the intervention groups was  **1.68 standard deviations lower**  (2.64 to 0.71 lower) |  | 758  (7 studies) | ⊕⊕⊝⊝  **low** | SMD -1.68 (-2.64 to -0.71) |
| **IL-1** | | | | | | | |
|  | GXST capsule Combined with Conventional Western Medicine/Conventional Western Medicine |  | The mean IL-1 in the intervention groups was  **1.39 standard deviations lower**  (2.37 to 0.41 lower) |  | 302  (3 studies) | ⊕⊝⊝⊝  **very low** | SMD -1.39 (-2.37 to -0.41) |
| **Whole blood viscosity** | | | | | | | |
|  | GXST capsule Combined with Conventional Western Medicine/Conventional Western Medicine |  | The mean whole blood viscosity in the intervention groups was  **0.55 lower**  (1.28 lower to 0.19 higher) |  | 408  (2 studies) | ⊕⊕⊝⊝  **low** |  |
| **Plasma viscosity** | | | | | | | |
|  | GXST capsule Combined with Conventional Western Medicine/Conventional Western Medicine |  | The mean plasma viscosity in the intervention groups was  **0.36 lower**  (0.56 to 0.16 lower) |  | 489  (3 studies) | ⊕⊝⊝⊝  **very low** |  |
| **LVEF** | | | | | | | |
|  | GXST capsule Combined with Conventional Western Medicine/Conventional Western Medicine |  | The mean LVEF in the intervention groups was  **4.86 higher**  (0.7 to 9.01 higher) |  | 177  (2 studies) | ⊕⊕⊝⊝  **low** |  |
| **Incidence of adverse reactions** | | | | | | | |
|  | GXST capsule Combined with Conventional Western Medicine/Conventional Western Medicine | **Study population** | | **OR 0.89**  (0.47 to 1.71) | 1083  (9 studies) | ⊕⊕⊝⊝  **low** |  |
|  |  | **145 per 1000** | **131 per 1000**  (74 to 224) |  |  |  |  |
|  |  | **Moderate** | |  |  |  |  |
|  |  | **97 per 1000** | **87 per 1000**  (48 to 155) |  |  |  |  |

**Supplementary File S4. （Subgroup analysis on the types of angina pectoris）**

**
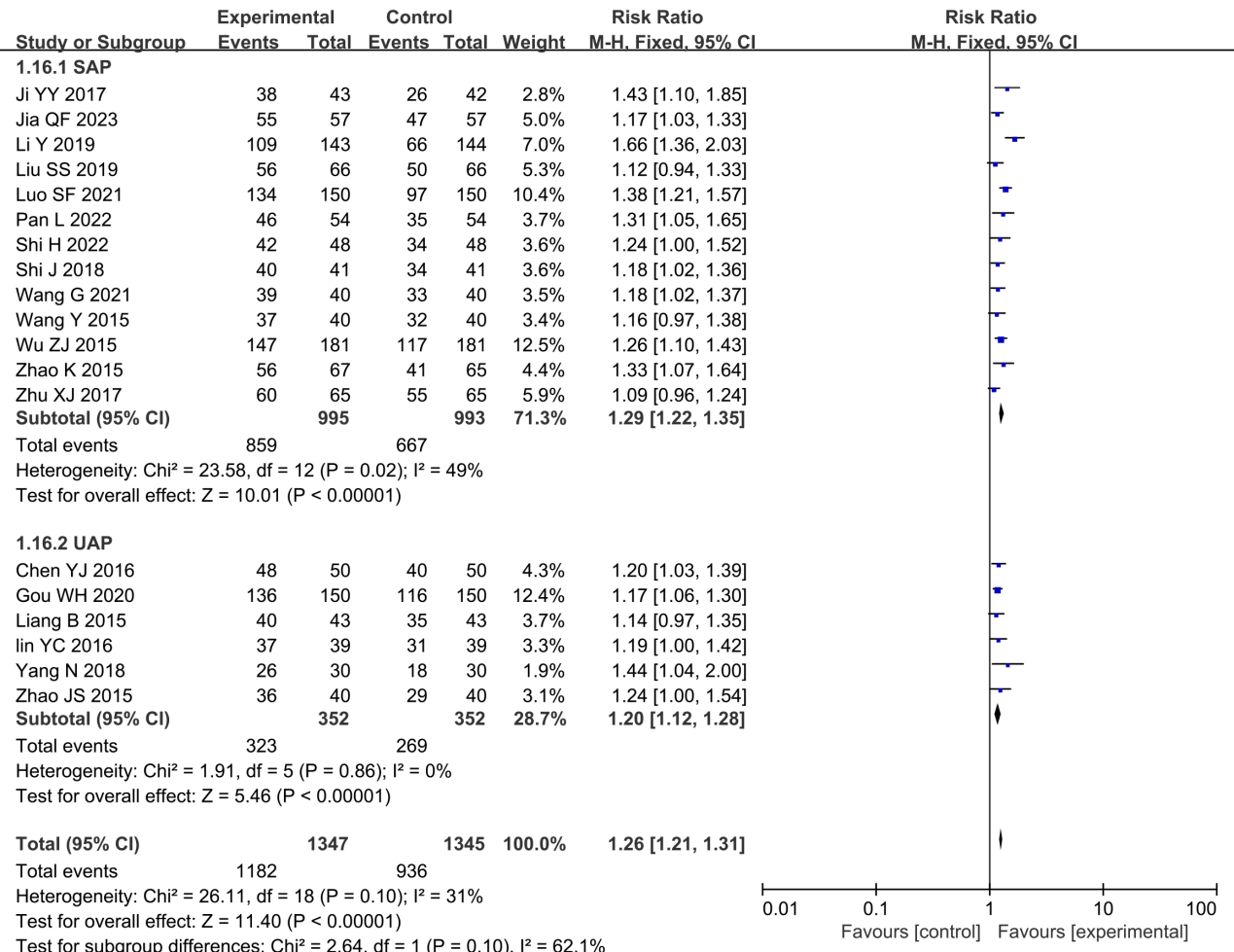
**

FIGURE 1 Total effective rate of angina pectoris


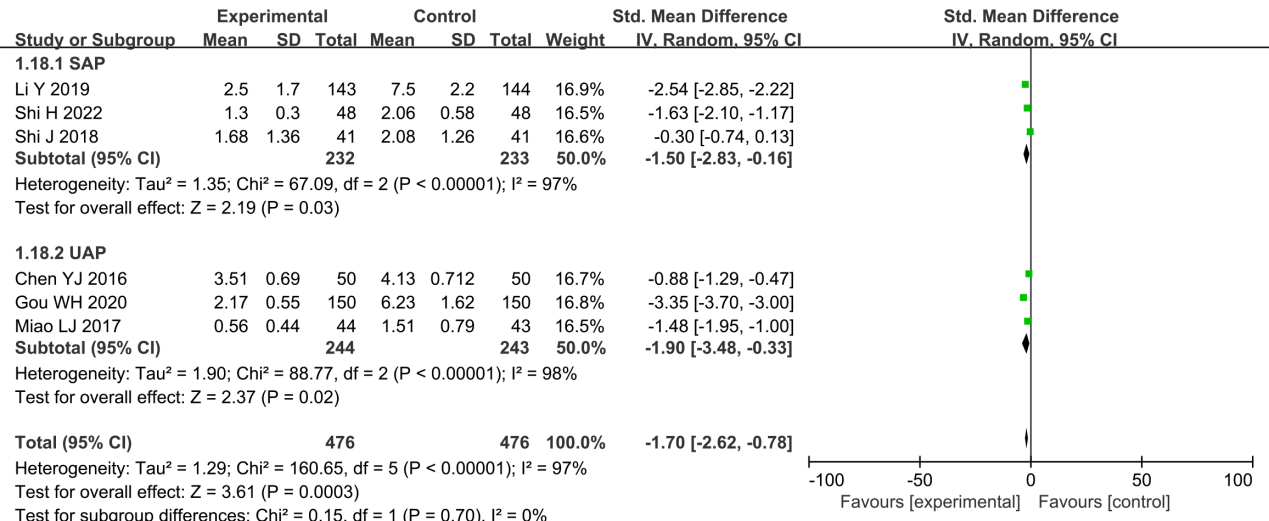


FIGURE 2 Frequency of angina pectoris


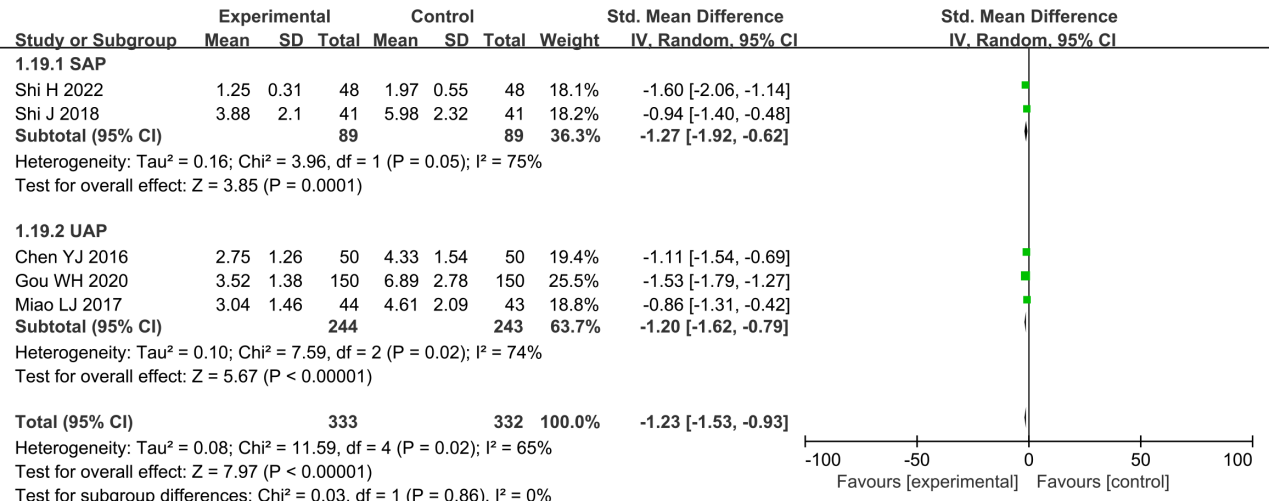


FIGURE 3 Duration of angina pectoris


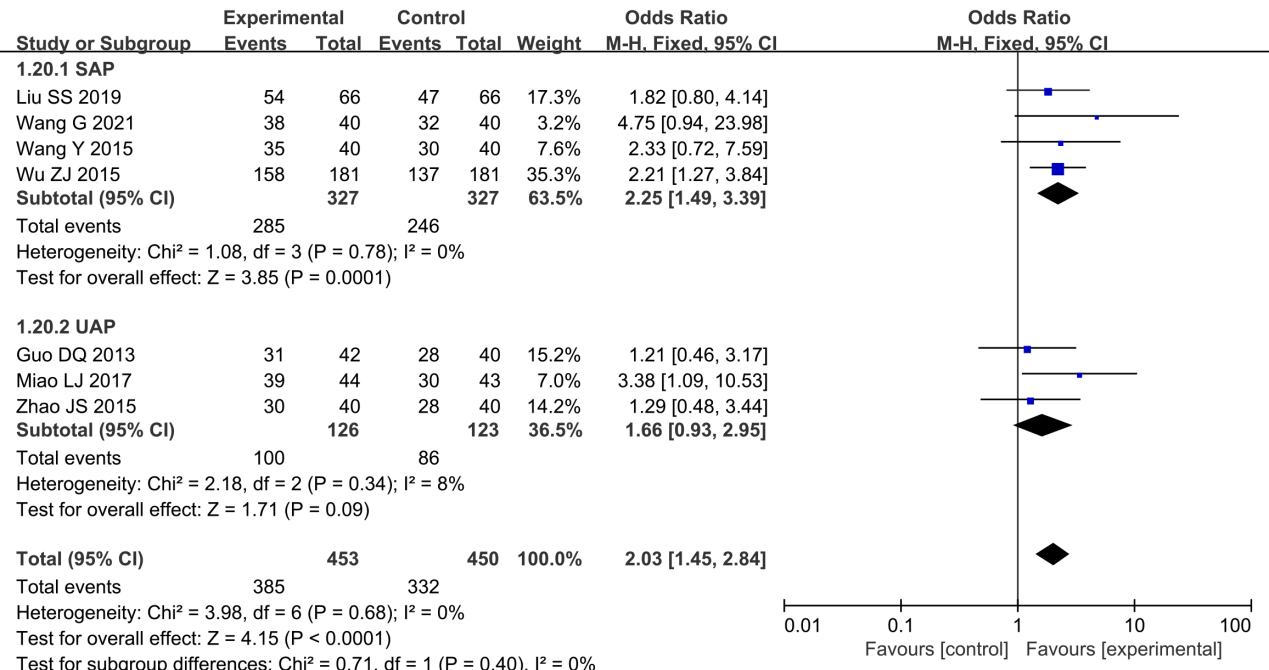


FIGURE 4 Total effective rate of ECG


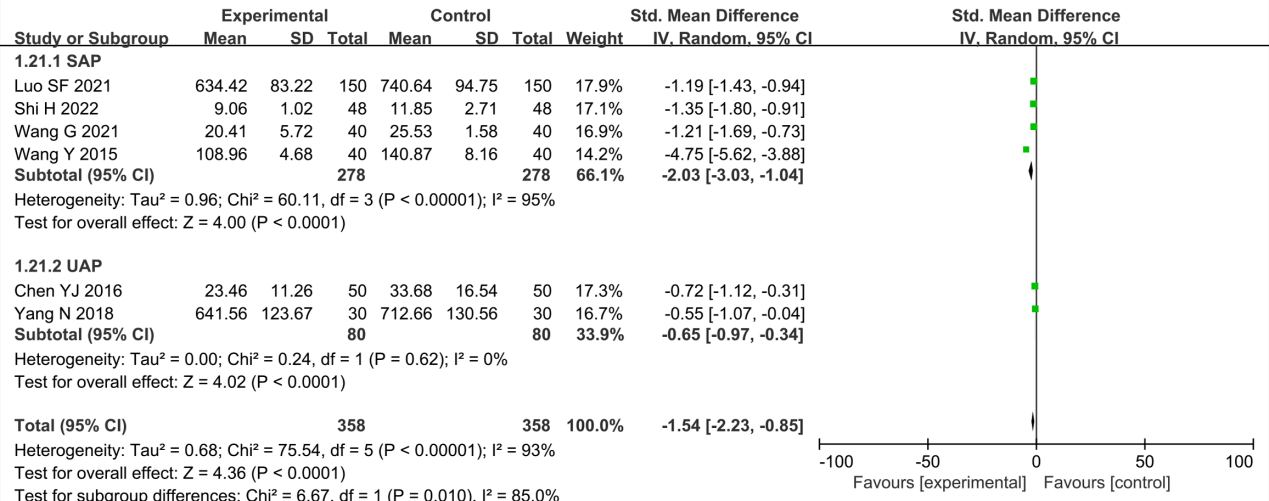


FIGURE5 IL-1


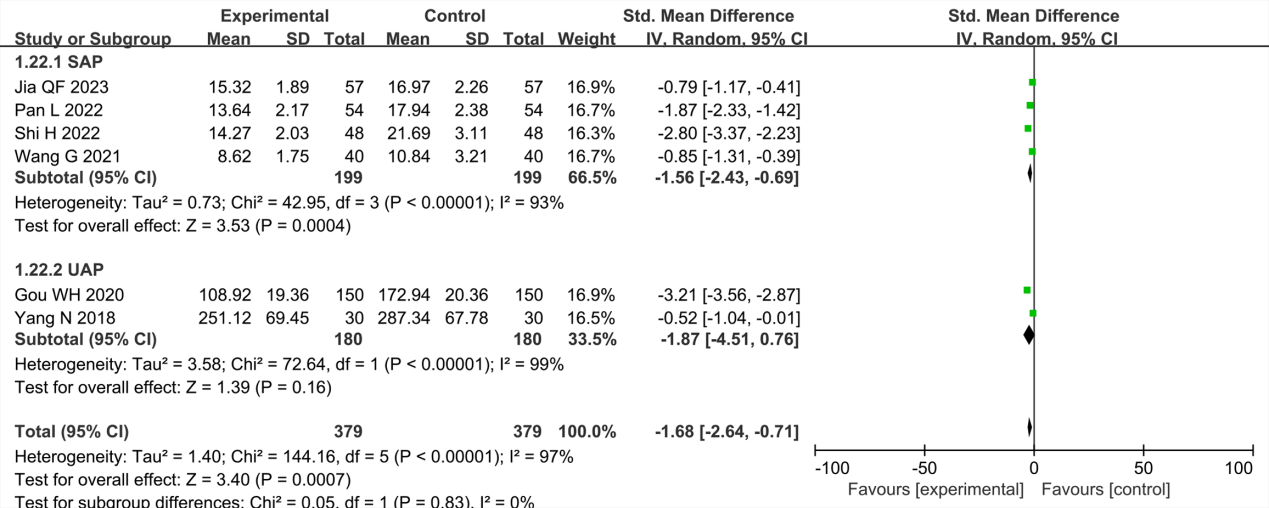


FIGURE 6 TNF-α


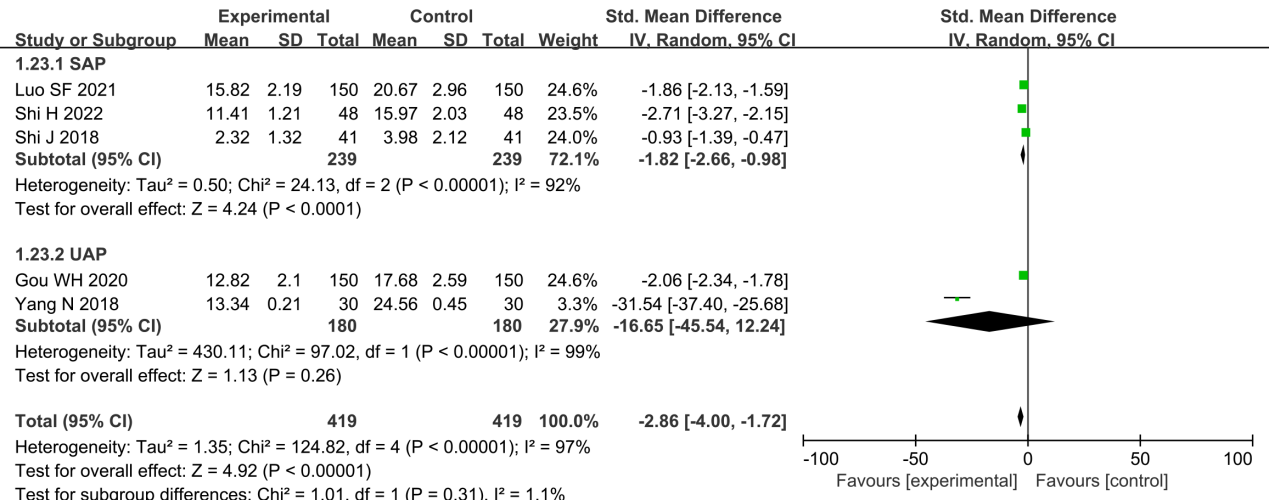


FIGURE 7 hs-CRP


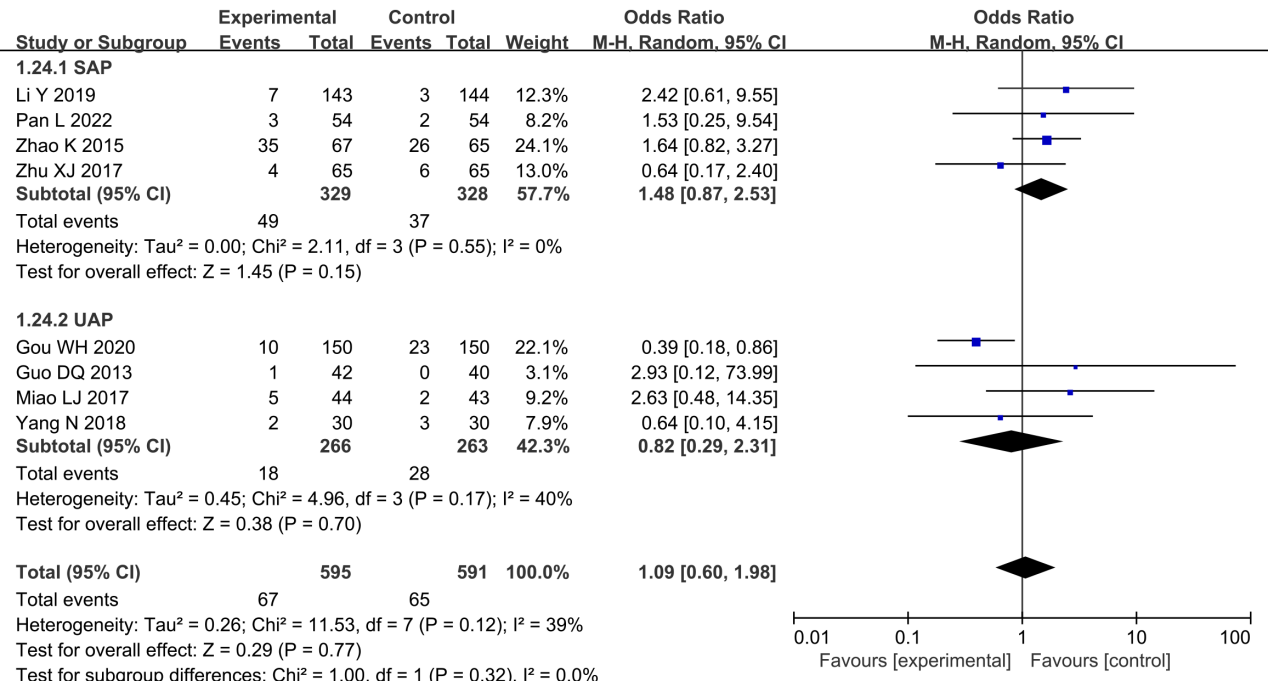


FIGURE 8 Adverse reactions.
